# Characterization of the virus-host RNA-RNA interactome across important human pathogenic RNA viruses

**DOI:** 10.1371/journal.ppat.1014217

**Published:** 2026-05-15

**Authors:** Nkerorema Djodji Damas, Stefan E. Seemann, Rui Costa, Janina Krambrich, Nicolas Fossat, Lizandro R. Rivera-Rangel, Ulrik Fahnøe, Louise Nielsen, Jan Gorodkin, Jens Bukh, Troels K. H. Scheel

**Affiliations:** 1 Copenhagen Hepatitis C Program (CO-HEP), Department of Infectious Diseases, Hvidovre Hospital and Department of Immunology and Microbiology, University of Copenhagen, Copenhagen, Denmark; 2 Center for non-coding RNA in Technology and Health, University of Copenhagen, Copenhagen, Denmark; 3 Department of Veterinary and Animal Sciences, University of Copenhagen, Copenhagen, Denmark; 4 Department for Molecular and Medical Virology, Ruhr University Bochum, Bochum, Germany; 5 Laboratory of Virology and Infectious Disease, The Rockefeller University, New York, New York, United States of America; Stanford University, UNITED STATES OF AMERICA

## Abstract

Viruses make extensive use of host cell machinery, however, most systematic studies of virus-host interactions focused on proteins with less attention to nucleic acids. RNA plays important roles in storing, conveying, and regulating genetic information. Our understanding of functional interactions between viral and host RNA is dominated by interactions with micro-RNAs (miRNAs), such as the interaction between hepatitis C virus (HCV) and the liver specific miR-122, critical for viral replication. Methodological developments, however, allow for broader exploration of the RNA interaction landscape beyond that of miRNAs. We here set out to identify virus-host RNA interactions by optimizing RNA antisense purification to systematically map RNA-RNA interactions (RAP-RNA) for viral RNA. After using the HCV/miR-122 interaction for validation, we applied RAP-RNA to determine the RNA interactomes for three important human pathogens; HCV, yellow fever virus (YFV) and chikungunya virus (CHIKV). Comparing virus-host RNA interactomes, we observed patterns of mRNAs encoding factors involved in translation and the proteasome, mitochondrial (mt)RNAs, small nucleolar (sno)RNAs and small nuclear (sn)RNAs, thereby providing a more comprehensive understanding of cellular RNA interactions for RNA viruses. Experimental targeting of selected HCV interactors did not lead to significant impact on viral infection, suggesting that the majority of virus-host RNA interactions are not critical for the virus. In contrast, transcription data were consistent with a possible role in viral stabilization of host RNA interactors. These findings may guide future research directions, e.g., for the role of snoRNAs and snRNAs in viral RNA regulation, with potential to provide further insight to viral exploitation of host factors.

## Author summary

As intracellular pathogens, viruses depend on their host cells and interact with a number of cellular molecules. Understanding these interactions is important, e.g., as they can serve as antiviral targets. Whereas viral interaction with cellular proteins is relatively well described, interaction with cellular RNA is much less understood. We here map interactions between viral and cellular RNA during infection with three important human pathogens; hepatitis C virus (HCV), yellow fever virus (YFV), and chikungunya virus (CHIKV). The blood borne HCV can lead to chronic infection of the liver and associated liver cancer. YFV and CHIKV are mosquito borne viruses that can lead to febrile illness followed by liver disease and chronic arthritis, respectively. Our findings reveal interactions with cellular RNA molecules involved in a number of processes, including modification and regulation of cellular RNA metabolism with potential to serve as antiviral targets.

## Introduction

Viral infections are responsible for significant morbidity and mortality worldwide. Thorough understanding of basic virology is critical for informed development of prevention and control. As obligate intracellular pathogens, viruses make extensive use of host cell machinery. The interfaces between host and virus have provided extraordinary insights to molecular mechanisms of pathogenesis and yielded valuable therapeutic targets. Furthermore, virology provided numerous tools and basic discoveries in cell biology [1].

Most systematic studies of virus-host interactions, however, have focused on proteins with less attention to nucleic acids. RNA mediates numerous functions in storing, conveying and regulating genetic information, and RNA-centric approaches to therapy are accelerating [2]. Among the relatively few well-characterized functional interactions between viral and host RNA [3] are micro-RNA (miRNA) interactions. Bound by argonaute (AGO) proteins of the RNA induced silencing complex (RISC), miRNAs in their canonical function bind 3’ UTRs of messenger RNAs (mRNAs) to down-regulate their expression [4]. Important examples of cellular miRNAs targeting RNA viruses include Eastern equine encephalitis virus (EEEV), an emerging mosquito-borne alphavirus, which is repressed by miR-142-3p binding to its 3’UTR. This restricts replication and evades interferon production specifically in antigen presenting cells, thereby allowing the virus to partially escape the immune system [5]. Contrarily, hepatitis C virus (HCV) critically depends on direct interactions between the liver specific miR-122-5p and two binding sites in the viral 5’ UTR [6,7]. Unlike for mRNAs, this interaction stimulates translation [8], and stabilizes and protects the HCV RNA genome from degradation [9–11]. Similar functions were described for the interaction between the miR-17-5p family and the 3’ UTR of bovine viral diarrhea virus (BVDV) [12,13]. The dependence of retroviruses on transfer RNAs (tRNAs) to prime replication constitutes another example of virus-host RNA interactions [14]. Furthermore, cellular non-coding RNAs (ncRNAs) engage in diverse RNA interactions, and some of these are emerging drug targets [15]. Hence, it is conceivable that many important virus-host interactions at the RNA interface await discovery.

Interactions between RNA and RNA-binding proteins (RBPs) are identified by deep sequencing based technologies like cross-linking immunoprecipitation (CLIP) [16,17], and the mass-spectrometry (MS) based RNA interactome capture and related methods [18–21] as well as RNA antisense precipitation coupled with MS (RAP-MS) [22]. Methods like RAP-RNA [23], sequencing of psoralen crosslinked, ligated, and selected hybrids (SPLASH) [24], and cross-linking of matched RNAs and deep sequencing (COMRADES) [25], identify direct RNA-RNA interactions. More recently, these methodologies have also been applied to virus infected cells, mapping RNA-RNA interactions for dengue virus (DENV), Zika virus (ZIKV) [25,26], severe acute respiratory syndrome coronavirus 2 (SARS-CoV-2) [27,28], respiratory syncytial virus (RSV) and vesicular stomatitis virus (VSV) [29]. These studies have identified interactors among small nuclear RNAs (snRNAs), such as U-RNAs, small nucleolar RNAs (snoRNAs), mRNAs and mitochondrial RNA (mtRNA). For many such interactors, functional roles have not been identified, although a pro-viral role for the miR-21 and 7SK RNAs and an anti-viral role for DYNLT mRNA was identified among ZIKV interactors [25,26]. Furthermore, blocking of the RSV mRNA interactors, KANK2 and CD44, led to reduced infection levels [29].

In the current study, we set out to characterize the broader RNA binding landscape for HCV. HCV is an important human pathogen, chronically infecting ~50 million people, leading to severe liver disease including fibrosis, cirrhosis, and hepatocellular carcinoma [30,31]. Although inhibitors of the HCV/miR-122-5p interaction demonstrated good efficacy in clinical trials as first-in-class therapeutics [32,33], these have not come to the market due to the success of small molecule directly acting antivirals (DAAs), which lead to sustained viral response rates in >95% of patients [34,35]. Nonetheless, the successful outcome of miR-122-5p inhibitors in clinical trials holds promise for RNA-based therapeutics against other targets during viral infection. No vaccine is available for HCV, and much remains to be understood regarding immune responses and pathology [36].

We here optimized and established RAP-RNA on HCV RNA and identified miR-122-5p as a positive control. This allowed us to globally map the most significant RNA interactors for HCV, and the function of selected candidates was studied. To demonstrate broader application of viral RAP-RNA and to better understand shared types of RNA interactors, we further performed RAP-RNA on cells infected with yellow fever virus (YFV) or chikungunya virus (CHIKV), both important mosquito-borne human pathogens [37,38]. YFV is an orthoflavivirus related to DENV and ZIKV, allowing comparison to previously published data, whereas the CHIKV interactome furthers the understanding of RNA-interactions for alphaviruses. Global comparison of interactome profiles within this study and with published datasets for other viruses allowed identification of shared interactor clusters.

## Results

### Establishment of virus-specific RAP-RNA

**Fig 1:**
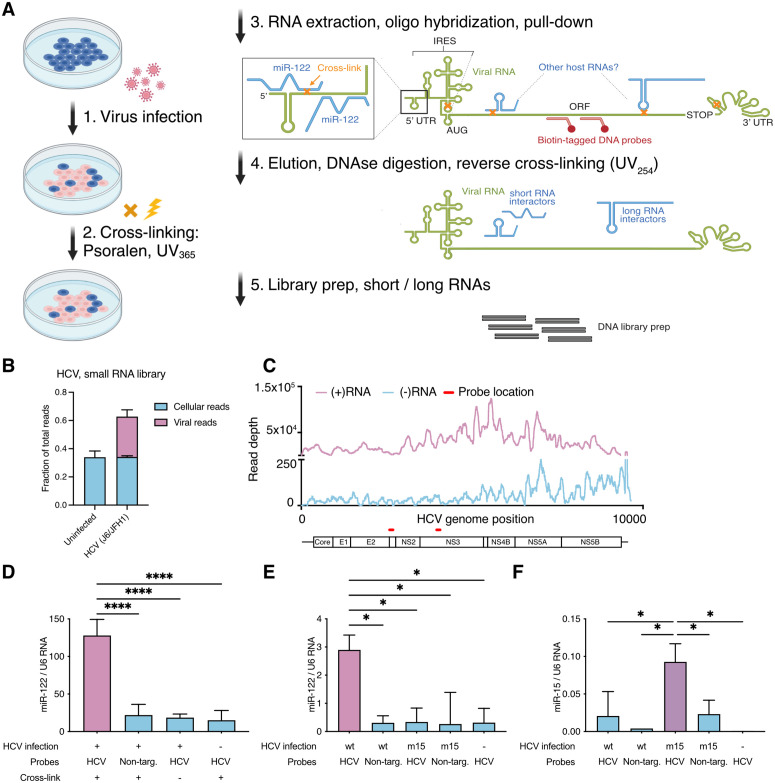
Establishment of RAP-RNA for viral RNA. **A.** Schematic of the major steps in RAP-RNA applied to viral RNA. **B.** Fraction of reads from HCV RAP-RNA small RNA library mapping to HCV and the human genome. Unmapped and multi-mapping reads were excluded and are not shown. **C.** Coverage plot of HCV RAP-RNA reads across the HCV (+) and (–) strand RNA. Locations of the probes used for pull-down on the viral genome are indicated. **D.** After RNA pull-down, enrichment of miR-122 was assessed and normalized to U6 RNA levels. Non-targeting probes were designed against the YFV genome. **E-F.** Enrichment of miR-122 (E) and miR-15 (F) as in **(D)**, but comparing HCV J6/JFH1-c2 with wild-type miR-122 seed sites (HCVwt) to miR-15 seed sites (HCVm15). In **(D-F)**, ANOVA with Dunnett correction for multiple testing was used for statistics. *: p < 0.033, **: p < 0.002, ***: p < 0.0002, ****: p < 0.0001. Parts of panel A were created in BioRender. Scheel, **T.** (2026) https://BioRender.com/2vxy5ut.

To identify cellular RNA molecules interacting directly with viral RNA, we modified the RNA antisense purification method for RNA (RAP-RNA) [23]. The original RAP-RNA method requires an elaborate and expensive probe tiling across the entire RNA molecule of interest. To simplify this, we instead pursued pull-down of viral RNA using few specifically optimized ~100 nucleotide biotinylated DNA probes. The probe hybridization efficacy was determined using RNAse H cleavage assays (S1A Fig) [39], and the most efficient probes were selected for use in RAP-RNA (Fig 1A). Briefly, in the modified RAP-RNA protocol, RNA molecules in close proximity were cross-linked using UV_365_ in the presence of the psoralen derivative, 4′-aminomethyltrioxsalen (AMT). Cells were then fractionated into nuclear and cytoplasmic constituents, and cytoplasmic viral RNA was enriched using pull-down with specific DNA probes. The enriched RNA was then DNAse treated to remove the DNA probes, and reverse cross-linked using UV_254_. To allow efficient identification of both short and long RNA interactors, two parallel strand-specific RNA-library methods were applied, based on poly-adenylation and template switching, or random hexamer priming, respectively.

To validate viral RAP-RNA, we targeted genomic HCV (+)RNA from Huh-7.5 hepatoma cells infected with the genotype 2a recombinant, J6/JFH1-c2 (72 hpi, MOI = 1) [40], using probes located in the p7 and NS3 regions. RAP-RNA identified around 50% of RNA reads mapping to HCV with the majority representing the targeted HCV (+)RNA (Fig 1B). As expected from active replication complexes or dsRNA, we also captured HCV (–)RNA. This was primarily (–)RNA reverse complementary to the (+)RNA downstream of the capture probes, consistent with probe inaccessibility for dsRNA extending throughout the p7-NS3 region (Fig 1C). Accordingly, specific RNA pull-down and identification of intermolecular RNA interactions through cross-linking could be confirmed.

To validate RAP-RNA identification of intermolecular RNA interactions, we analyzed the well-characterized HCV interactor, miR-122-5p. Using miRNA specific RT-qPCR, we found miR-122-5p specifically enriched upon pull-down with HCV-specific probes from cross-linked, infected cells, compared to pull-down with non-targeting probes, non-cross-linked, or uninfected cells (Fig 1D). To corroborate the specificity of this finding, we repeated this experiment with the previously described HCV-m15 mutant, for which the miR-122-5p binding sites are replaced with those of miR-15-5p. This mutant binds and requires miR-15-5p/miR-16-5p, and not miR-122-5p, for viral replication, and is slightly attenuated [12,41]. For HCV-m15, RNA pull-down led to enrichment of miR-15-5p and not miR-122-5p (Fig 1E-F). This demonstrated that RAP-RNA identifies intermolecular interactions between the targeted viral RNA and specific viral and cellular RNA interactors.

### Mapping interactions between HCV RNA and short cellular RNAs

**Fig 2:**
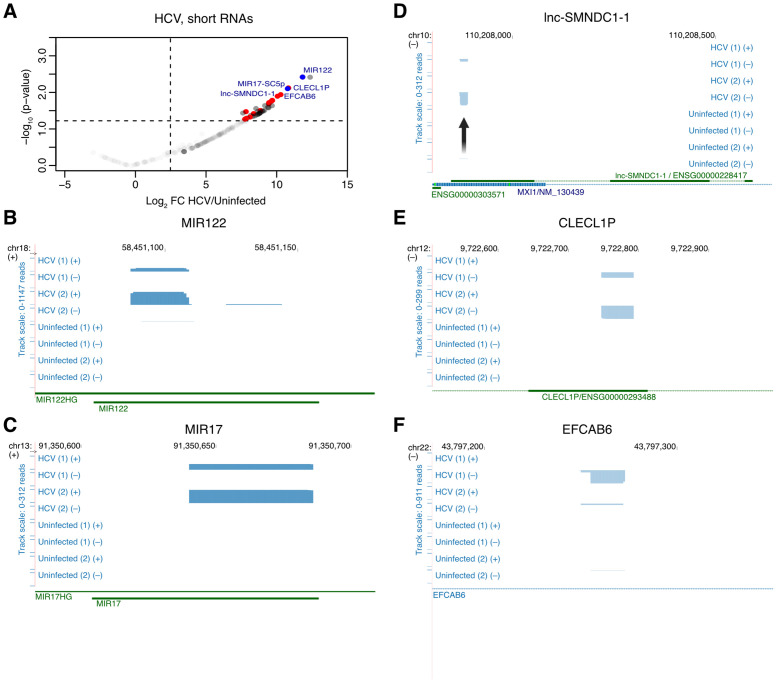
Global identification of HCV interactors from the small RNA library. **A.** Volcano plot of RAP-RNA for HCV small RNA library showing –log_10_(p-value) as a function of the log_2_ fold change enrichment in HCV infected compared to uninfected samples. Dotted lines indicate significance thresholds of p < 0.05 and log_2_FC>2. Red dots indicate genes meeting these requirements and for which a significantly enriched peak was identified; MIR122 and MIR17-SC5p are shown in blue. The data is based on 2 RAP-RNA replicates. **B-F.** Genome coverage plots for the most enriched interactors, MIR122 **(B)**, MIR17 **(C)**, lnc-SMNDC1-1 **(D)**, CLECL1P **(E)** and EFCAB6 **(F)**. Chromosomal location and strand are indicated on the top. Strand-specific coverage plots are shown for the HCV infected and uninfected replicates. Track scale (identical for all tracks of the given panel) is indicated on the left. Refseq gene annotations are shown below. In **(D)**, an arrow indicates location of the peak.

We next globally analyzed small host RNAs enriched by HCV RAP-RNA compared to uninfected controls. Among candidate interactors, we set the following requirements: log_2_ fold enrichment (log_2_FC)>2, p < 0.05, observed in all infected libraries, and at least one significant peak identified in the candidate gene. This identified miR-122-5p, in addition to a miR-17 cleavage fragment, the long non-coding RNA lnc-SMNDC1–1 (ENSG00000228417), *EFCAB6* mRNA and *CLECL1P* pseudogene RNA, and 13 other mRNA interactors (Fig 2A and S1 Table). For miR-122, the identified reads as expected perfectly mapped to mature miR-122-5p (Fig 2B). The miR-17 cleavage fragment represented the left-over product from DICER single cleavage on the 5p strand of pre-miR-17 (m17-SC5p) (Fig 2C and S2A Fig). Specific binding peaks were observed on lnc-SMNDC1–1, antisense to the 5’ end of the *MXI1* gene (Fig 2D), and on *CLECL1P* exon 2 (Fig 2E), putatively representing HCV RNA interaction sites. Despite presence of a significant peak for *EFCAB6*, scattered reads mapping to the same RNA were also observed in control libraries (Fig 2F).

### Mapping interactions between HCV RNA and long cellular RNAs

**Fig 3:**
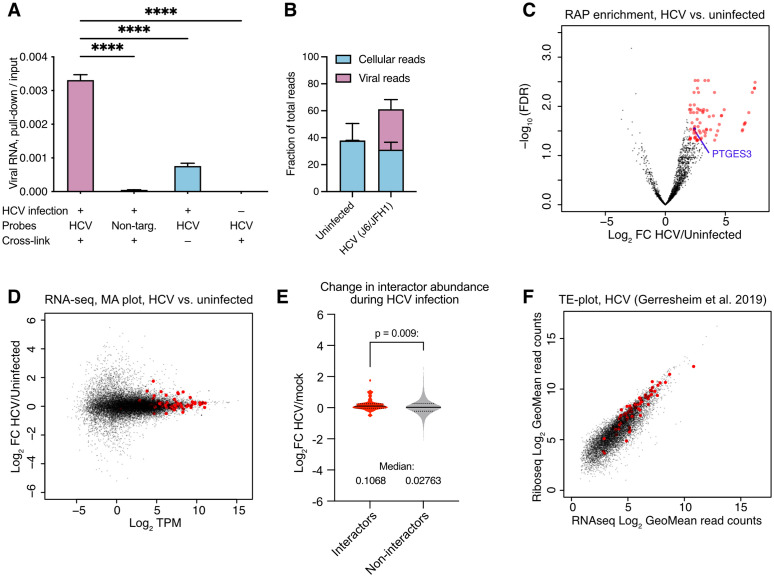
Global identification of HCV interactors from the long RNA library. **A.** Assessment of enrichment of viral RNA after pull-down compared to input. Values are given as fraction of pulled-down viral RNA compared to input level. Non-specific probes were targeting YFV. ANOVA with Dunnett correction for multiple testing was used for statistics. *: p < 0.033, **: p < 0.002, ***: p < 0.0002, ****: p < 0.0001 **B.** Fraction of reads from HCV RAP-RNA long RNA library mapping to HCV and the human genome. Unmapped and multi-mapping reads were excluded and are not shown. **C.** Volcano plot of RAP-RNA for HCV long RNA library showing –log_10_(FDR) as a function of the log_2_ fold change enrichment for HCV infected compared to uninfected samples. Red dots indicate genes meeting significance thresholds of FDR < 0.05 and log_2_FC>2. The data is based on 6 RAP-RNA replicates. PTGES3 is indicated in blue. **D.** MA plot of transcriptomics data from a parallel experiment with identical infection conditions [41], showing the log_2_FC for HCV infected compared to uninfected samples as a function of gene abundance (TPM: transcripts per million). The 74 significant HCV RNA interactors as identified in RAP-RNA are highlighted in red. **E.** Log_2_FC in interactor abundance during HCV infection compared to non-interactors. Mann-Whitney U-test was used for statistics. **F.** Translation efficiency (TE) plot showing Log_2_ GeoMean read counts from ribosome profiling (translation activity) as a function of that of RNA-seq (transcriptomic activity) from HCV Jc1 infection of Huh-7.5 cells [43]. The 74 significant HCV RNA interactors as identified in RAP-RNA are highlighted in red.

To validate the long RNA library protocol, we also applied this to HCV infected Huh-7.5 cells (J6/JFH1-c2, 72 hpi, MOI = 1) and confirmed probe- and infection-specific pull-down of viral RNA using RT-qPCR (Fig 3A). Continuing with library preparation and high-throughput sequencing, we identified ~30% viral reads and another ~30% reads mapping to the host cell genome (Fig 3B). We identified 74 cellular RNAs that were significantly enriched for the infected samples (log_2_FC>2 and FDR < 0.05) (Fig 3C). These included mRNAs encoding for several members of the ribosome and other translation factors (e.g., *EEF2, EIF1, and EIF4A*), molecular chaperones (*CCT2*, *3* and *8*), ATP synthase (*ATP5F1*), proteasome (*PSMC3, 4* and *7*), and splicing factors (*SRSF2, 7 and 9*) (S1 Table). Although low-abundant RNAs were generally not captured in HCV RAP, the 74 significant RNA interactors represented a broad range of expression levels during J6/JFH1-c2 infection of Huh-7.5 cells, as determined by transcriptomic analysis (Fig 3D and S1B Fig) [41]. Accordingly, interaction with viral RNA was not merely a matter of intracellular abundance. On average, the abundance of the 74 HCV RNA interactors was slightly but significantly increased upon infection compared to non-interactors (Fig 3E). This observation is in agreement with a recent study on SARS-CoV-2, suggesting that interactions with viral RNA may stabilize mRNAs during infection [42]. We furthermore hypothesized that highly translated RNAs could be overrepresented among interactors, as such could have a higher likelihood to locate to the vicinity of viral RNA during translation. To investigate this, we analyzed a published dataset combining ribosome profiling and transcriptomics during infection with the closely related HCV Jc1 variant in Huh-7.5 cells [43]. In this dataset, RNA abundance and corresponding translational level were highly correlated. The 74 HCV interactors identified by RAP-RNA were broadly represented in the spectrum of translational efficiencies (Fig 3F). In fact, their translational efficiency was slightly lower compared to all other mRNAs (S1C Fig). This difference, however, could be an effect of the higher divergence in translational efficiency (TE) among low-abundant RNAs, which were not represented among the HCV RNA interactors.

Finally, we performed gene ontology analysis on the 74 HCV RNA interactors but found no significantly enriched terms. Nevertheless, the specific identified cellular RNA interactors suggested a potential role for RNA-RNA interactions during HCV infection in regulating posttranscriptional processing at the RNA level (splicing) or the protein level (translation, protein folding, protein turnover).

### Functional analysis of selected interactors

**Fig 4:**
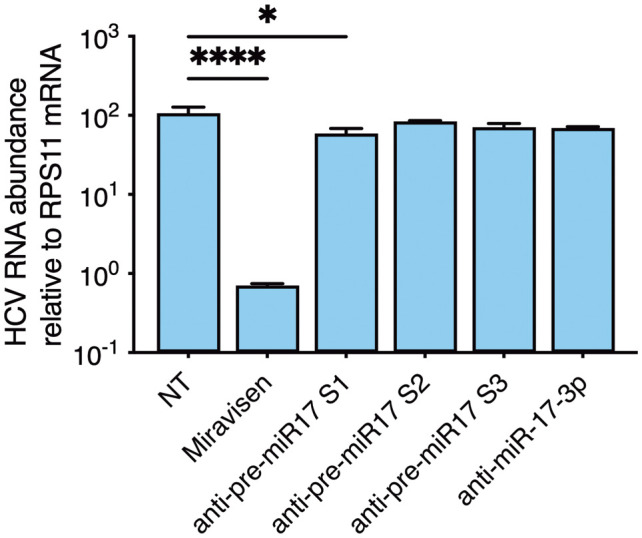
Analysis of the role of m17-SC5p during HCV replication. HCV replication during LNA treatment as measured by RT-qPCR relative to the house keeping gene RPS11. NT: Non-targeting control. Miravirsen is a miR-122 LNA inhibitor with effect against HCV replication as demonstrated in vitro and in clinical trials. The regions of pre-miR-17 targeted by the specific LNAs are indicated in S2 Fig. ANOVA with Dunnett correction for multiple testing was used for statistics. *: p < 0.033, **: p < 0.002, ***: p < 0.0002, ****: p < 0.0001.

To next investigate whether specific HCV RNA interactors would play functional roles during infection, we initially selected the m17-SC5p and prostaglandin E synthase 3 (*PTGES3*) mRNAs (marked in blue, Figs 2A and 3C) from the short and long RNA pipelines, respectively, for further analysis. To investigate whether m17-SC5p was important for HCV replication, we designed LNA inhibitors targeting pre-miR-17 regions inside and outside the m17-SC5p fragment (S2A Fig). Upon transfection into Huh-7.5 cells prior to infection with HCV, the well-described miR-122 LNA inhibitor [44], miravirsen, led to >100-fold reduction in HCV RNA levels (Fig 4). No effect was observed for the m17-SC5p targeting LNAs, whereas a significant but minor reduction was observed for anti-pre-miR17-S1, targeting pre-miR-17 outside of the m17-SC5p region. As a control, we confirmed that none of the antisense inhibitors reduced abundance of the pri-miRNA forms (S2B-C Fig). RNA interaction prediction led to a number of possible interaction sites on both m17-SC5p and on the HCV RNA in particular, challenging site directed mutagenesis studies. We therefore interrogated published AGO-CLIP data sets from HCV infected cells [12,41] for presence of the m17-SC5p sequence in chimeric form with viral sequence, however, without identifying such sequences. Accordingly, this did not lend support towards AGO being involved in the interaction between m17-SC5p and HCV RNA and did not allow us to further identify the interaction site on the viral RNA. In aggregate, despite identification of a direct RNA-RNA interaction, we could not identify a functional pro- or antiviral role of m17-SC5p for HCV replication.

**Fig 5:**
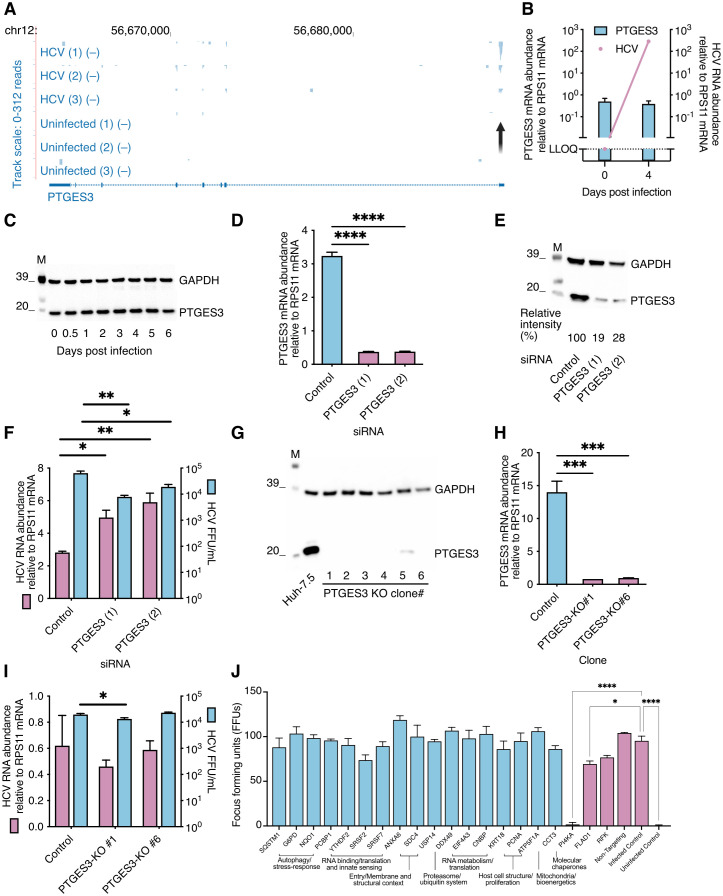
Analysis of the role of mRNA interactors for HCV infection. **A.** HCV RAP-RNA genome coverage plot for PTGES3. Chromosomal location is indicated on the top. Strand-specific coverage plots are shown for the HCV infected and uninfected replicates. Track scale (identical for all tracks) is indicated on the left. Refseq gene annotation is shown below. An arrow indicates location of the peak. **B.** Quantification of HCV RNA and PTGES3 mRNA by RT-qPCR 4 days after HCV J6/JFH-c2 infection. Normalized to the house keeping gene RPS11. **C.** Quantification of PTGES3 protein levels by Western blotting after HCV infection. House keeping protein GAPDH is shown as control. **D-E.** Quantification of PTGES3 mRNA by RT-qPCR (D) or protein by Western blotting (E) after transfection with two different siRNAs targeting PTGES3. **F.** Quantification of HCV J6/JFH1-c2 replication by RT-qPCR or infectious virus production by focus-forming unit (FFU) assay after transfection with PTGES3 siRNA. **G.** Analysis of PTGES3 protein levels by Western blotting in six different Huh-7.5 CRISPR knock-out (KO) clones. **H.** Quantification of PTGES3 mRNA by RT-qPCR in KO clones #1 and #6. **I.** Quantification of HCV J6/JFH1-c2 replication by RT-qPCR or infectious virus production by FFU assay 72 hrs after infection of parental Huh-7.5 cells or KO clones #1 and #6. **J.** HCV J6/JFH1-c2 infection as quantified by FFU count 48 hours post infection of cells treated prior with siRNAs of the indicated mRNAs. The inoculum was titrated to ~100 FFUs in pilot experiments. Tested candidates are shown in light blue, and known host factors and controls in dusty pink. Functional categories are indicated below for the tested candidates. In (B), two-tailed t-test and in (D, F, H, I and J) ANOVA with Dunnett correction for multiple testing was used for statistics. ns: non-significant, *: p < 0.033, **: p < 0.002, ***: p < 0.0002, ****: p < 0.0001.

The mRNA interactor, *PTGES3*, had a representative enrichment level (Fig 3C) and a highly enriched peak on the mRNA 5’ UTR (Fig 5A). We first determined whether HCV infection regulated *PTGES3* expression at the transcriptional or translational level; no changes to *PTGES3* levels were observed (Fig 5B-C). Knocking down *PTGES3* mRNA using two independent siRNAs led to a 5–10-fold reduction in mRNA and protein levels (Fig 5D-E). Compared to a control, HCV infection under these conditions led to a 2-fold increase in intracellular viral RNA levels, combined with ~5-fold decrease in extracellular infectivity titers (Fig 5F). We finally used CRISPR/Cas to knock out *PTGES3.* This led to complete or almost complete abrogation of protein expression in six analyzed cell clones (Fig 5G). Clones #1 and #6 were expanded to allow further studies, and for both, the *PTGES3* mRNA level was reduced ~10-fold (Fig 5H). Compared to parental Huh-7.5 cells, HCV replication was similar in these clones, as determined by intracellular viral RNA levels (Fig 5I). A significant, but minimal reduction in virus production was observed for clone #1 but not #6. In aggregate, despite the direct RNA-RNA interaction, these data did not suggest a critical function for PTGES3 mRNA during the HCV replication cycle, although a minor supportive role on particle production cannot be excluded.

To explore whether other RNA interactors modulate HCV replication, we performed a focused siRNA loss-of-function screen of 17 additional HCV RNA interactors in Huh‑7.5 cells prior to infection with HCV J6/JFH1-c2. Interactors were selected to represent examples involved in autophagy and stress responses, RNA binding and translation, innate sensing, membrane and structural context, RNA metabolism and splicing, host cell structure and proliferation, the proteasome, mitochondrial bioenergetics, and molecular chaperoning. Whereas significant reduction in HCV infection was observed for the known host factors and controls, PI4KA [45] and FLAD1 [46,47], no significant effect was observed from knocking down any of the 17 RNA interactors (Fig 5J and S3 Fig). This suggested that most RNA interactors do not functionally affect viral infection.

### Mapping of RNA-RNA interactions across RNA viruses

**Fig 6:**
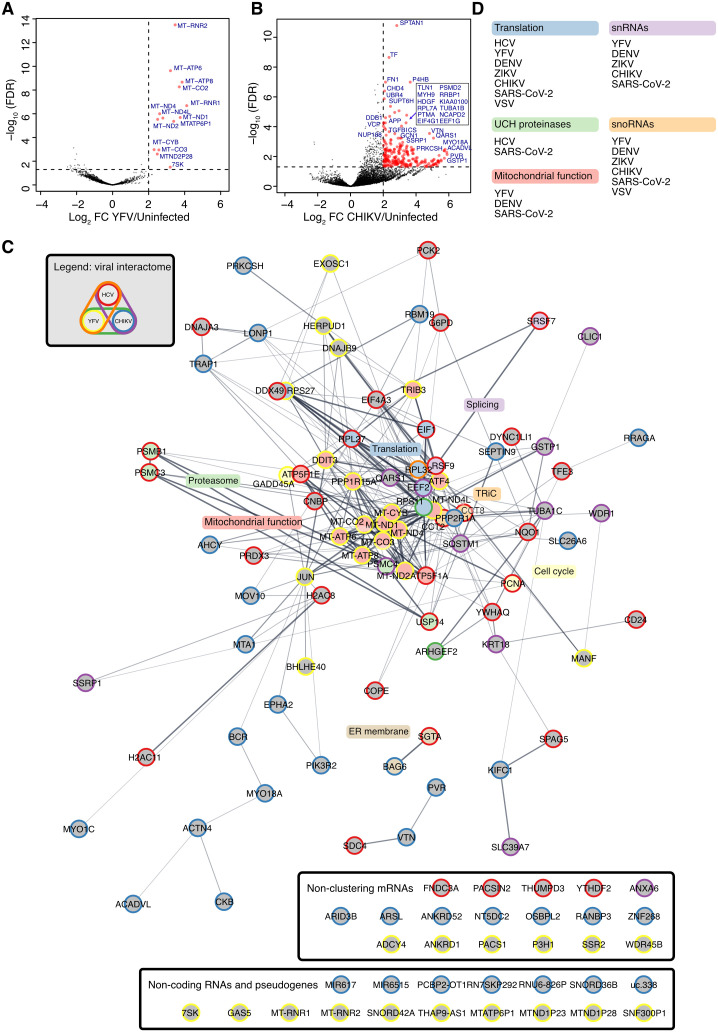
Analysis of RNA interactors across RNA viruses. **A.** Volcano plot of RAP-RNA for YFV RNA showing –log_10_(FDR) as a function of the log_2_ fold change enrichment in YFV infected compared to uninfected samples. Large red dots (with gene names) indicate genes meeting significance thresholds of FDR < 0.05 and log_2_FC>2. The data is based on 3 RAP-RNA replicates. **B.** Volcano plot as in (A) but for CHIKV RNA. Names are given only for selected, most significant interactors. The data is based on 3 RAP-RNA replicates. **C.** Clustering of HCV, YFV and CHIKV interactors based on interactions and shared function between derived protein products using the STRING database [48]. The 40 most enriched interactors (by log_2_FC) for each virus, as well as interactors with overlap between two viruses, were included. Node edges are colored according to viral interactome(s) as given in the legend. Node shading is according to functional annotation by DBSCAN clustering as given in colored boxes. TRiC: chaperonin-containing T-complex. Non-coding RNA and pseudogenes not represented in the STRING database were manually added and shown at the bottom. **D.** Grouping of RNA interactome data from the current study (HCV, YFV, CHIKV) and published studies (DENV, ZIKV, SARS-CoV-2, RSV and VSV) into general categories of RNA type and function (mRNAs).

To pursue further understanding of the HCV long RNA interactome and potential overlap with that of other viruses, we similarly performed RAP-RNA on Huh-7.5 cells infected with yellow fever virus (YFV, strain 17D, 24 hpi, MOI = 1), a related virus from the *Flaviviridae* family, or chikungunya virus (CHIKV, strain LR, 24 hpi, MOI = 1), an RNA virus from the related *Togaviridae* family. For YFV, we identified 15 cellular RNAs significantly enriched in RAP-RNA from the infected samples (log_2_FC>2 and FDR < 0.05), mostly mtRNAs as well as *RN7SK* (Fig 6A). Less stringent criteria (p-value<0.05) defined a list of 46 interactors, including a number of ribosomal mRNAs and snoRNAs (S1 Table). GO analysis of these 46 interactors revealed 12 significant terms of biological processes, all related to mitochondrial activity and oxidative phosphorylation.

For CHIKV, we identified 241 cellular RNAs significantly enriched in RAP-RNA from the infected sample (log_2_FC > 2 and FDR < 0.05) (Fig 6B and S1 Table). No significant terms were identified in GO analysis among the 241 interactors.

Between RNA interactors of HCV, YFV and CHIKV RNA, we in particular observed patterns of mRNAs encoding proteins involved in translational processes in the proteasome and in mitochondrial processes, as well as non-coding RNAs (Fig 6C). Among specific interactors, *RPL32* was shared between HCV and YFV, *ANXA6, CLIC1, EEF2, GSTP1, KRT18, PSMC4, SLC39A7, SQSTM1, SSRP1, TUBA1C, and WDR1* between HCV and CHIKV, and *ARHGEF2 and RPS11* between YFV and CHIKV. In a broader comparison of RNA interactors across different viruses, these findings corresponded to those of other orthoflaviviruses (DENV, ZIKV), where interactors were enriched among mRNAs encoding proteins involved with translational and RNA modification processes [26]. Specific mRNAs overlapping observations from the current study included *7SK*, *ACTB*, *CHD4, EIF1, GAPDH, PTMS, SNORA62, SNORD100,* a number of ribosomal mRNAs, as well as variants of dynein and tubulin components. SARS-CoV-2 RNA interactors shared with the current study comprise *7SK*, *APP, ARHGDIA, BHLHE40, CNBP, COPE, DDB1, DHCR7, DNAJA3, EPHA2, ESYT1, FASN, GANAB, GTF3C1, HERPUD1, KMT2D, NCAPD2, P4HB, PRDX1, PRKCSH, PSAP, PTBP1, SNORA21, SNORA62, SNORD100, SNRNP200, SQSTM1*, *TLN1*, as well as factors involved with mitochondrial and translational processes, the proteasome and structural components like those of actin pathways [28]. A recent study on VSV also identified snoRNAs as well as mRNA interactors involved in RNA processing, translation, and decay [29]. Thus, despite divergent RNA interactomes between RNA viruses, common patterns among RNA interactors emerge, including snRNAs, snoRNAs, and mRNAs involved in mitochondrial, translational and proteasome processes (Fig 6D).

## Discussion

Interaction between viral and cellular RNA has gained significant interest. In the current study, we optimized the RAP-RNA protocol to use a limited number of functionally validated probes, thereby making the method much more cost effective compared to the original probe tiling approach [23]. The optimized protocol was successfully applied to viral RNA, using the well-characterized HCV/miR-122 interaction to validate the approach. By further application, we used this to generate the first RNA-RNA interaction profiles for HCV, YFV and CHIKV.

Virus-miRNA interactions have been extensively investigated, in particular given the development of specific identification tools like crosslinking, ligation, and sequencing of hybrids (CLASH) [49,50] and covalent ligation of endogenous Argonaute-bound RNAs (CLEAR)-CLIP [12,51]. This led to identification of the critical interaction between the miR-17-5p family and pestiviruses, like BVDV [12,13]. Here, we confirmed the HCV/miR-122 interaction but also identified an interaction between HCV RNA and the m17-SC5p fragment. Whereas mature miRNAs can be important pro- or antiviral factors [3], the m17-SC5p represents a left-over product from DICER single cleavage on the 5p (SC5p) strand of the pre-miRNA. Such SC5p products were previously identified at 5–10% of DICER products and generally at higher frequencies compared to SC3p [52], although an earlier study observed higher SC3p compared to SC5p frequency specifically for miR-17 [53]. Despite their relatively high frequency among DICER products, their function remains unknown. Attempts to inhibit m17-SC5p using LNAs did not lead to profound effects on HCV replication, suggesting that this interaction may not have a major pro- or anti-viral impact. Alternatively, viral RNA could potentially interfere with miRNA processing, as was previously demonstrated for long non-coding RNA mediated inhibition of DROSHA cleavage [54].

An advantage of RAP-RNA is its ability to identify many RNA types beyond miRNAs. We here identified a number of mRNAs that exist in close proximity to viral RNA during infection. From a global perspective, we found that HCV RNA interactors were significantly enriched during infection compared to non-interactor RNAs. This finding would be consistent with mRNA stabilization through interaction with viral RNA, as previously suggested for SARS-CoV-2 [42]. In contrast, HCV mRNA interactors were not enriched among highly abundant or highly translating transcripts. Given its undescribed function in the HCV replication cycle, a representative enrichment level, and a highly enriched binding peak on its 5’ UTR, we selected the *PTGES3* mRNA for further functional studies. Knock-down experiments suggested a pro-viral role during assembly and release, however, this effect was at best minimal when studied in CRISPR-Cas KO clones. Interestingly, knock-down of additional 17 interactors did also not significantly impact HCV infection, whereas the parallel knock-down of known host factors did. This suggested that most HCV RNA interactions do not directly impact the viral life cycle. Furthermore, we found that instead of passively binding to the most abundant host transcripts, viral RNA specifically interacts with host mRNA with diverse functions. We speculate that interaction with specific RNAs indeed may be linked to the function of the encoded protein, in that following translation, the resulting protein will locate in immediate vicinity to the viral RNA. More broadly across different viruses, we found enrichment of mRNAs involved in translational processes, mitochondrial function, and of the proteasome, and this was also previously observed for other orthoflaviviruses and for SARS-CoV-2 (Fig 6C-D) [26,28]. Orthoflaviviruses like YFV are well known to regulate mitochondrial processes, including mitochondrial stress and potential release of mitochondrial nucleic acids [55], which further may explain the enrichment of mitochondrial RNAs among YFV and DENV [26] RNA interactors.

SnoRNAs primarily regulate RNA modifications, generally with C/D box snoRNAs regulating methylation and H/ACA box snoRNAs regulating pseudouridylation [56,57]. RNA modifications, such as m^6^A methylation, have been reported for HCV, flavi- and alphaviruses [58,59], although more recent studies could not confirm the presence of m^6^A methylation on flavi- and alphavirus RNA [60,61]. However, interaction between snoRNAs and viral RNA was previously reported for SARS-CoV-2, where it may mediate 2’O-methylation of the viral RNA [28], and also for DENV, ZIKV [26] and VSV [29]. Hence, snoRNA association with YFV and CHIKV RNA may suggest similar mechanisms of RNA modification, also given that snoRNA expression appears to be regulated during alphavirus infection [62].

SnRNAs are integral parts of the spliceosome. We here identified interactions between CHIKV and U4, and interaction with snRNAs were also previously reported for ZIKV [25] and for coronaviruses [27]. Although RNA viruses like CHIKV, ZIKV, and coronaviruses are not using canonical splicing, U1 was suggested to bind splice-site related motifs in the SARS-CoV-2 genome [27]. It would be possible that snRNAs may be hijacked by the virus to exert non-canonical functions akin to the function of miR-122 for HCV. Nonetheless, the cytoplasmic assembly of the Sm core small nuclear RNP [63] would provide an explanation for physical interaction of these otherwise nuclear RNAs with cytoplasmic RNA viruses.

In conclusion, this study established an approachable methodology for mapping of virus-host RNA-RNA interactions and confirmed known interactions. Limitations of RAP-RNA include (1) the continued requirement for probe design and optimization, although significantly diminished compared to the original protocol, and (2) the lack of information on the interaction site on the viral RNA. This is provided by methods like SPLASH [24], LIGR-Seq [64], PARIS [65] and COMRADES [25], and could have provided critical information to proceed with functional studies, e.g., for the HCV/m17-SC5 interaction. Nonetheless, the current study provides RNA interactome profiles for three important human viruses, HCV, YFV and CHIKV. Comparison of interactomes between these viruses and those of previous publications on DENV, ZIKV, SARS-CoV-2, RSV and VSV identified shared patterns of mRNAs encoding factors involved in translation and the proteasome, as well as mtRNAs, snoRNAs and snRNAs. The overlapping interactome profiles point to interesting future research directions, e.g., for the role of snoRNAs in modification of viral RNA or for possible viral hijacking of snRNAs. Such studies could yield even further insight to viral exploitation of cellular RNA molecules.

## Materials and methods

### Cell culture

Huh-7.5 hepatoma cells were maintained in Dulbecco’s Modified Medium (DMEM) containing L-glutamine supplemented with 10% fetal bovine serum (FBS) and 1% Penicillin/Streptomycin (PenStrep). BHK-J cells were cultured in Adenovirus Expression Medium (AEM) supplemented with 5% FBS and 2 mM Glutamine. All cell lines were maintained at 37 °C and 5% CO_2_.

### In-vitro transcription

HCV J6/JFH1 clone 2 RNA was obtained from a clone containing a T7 promoter at the 5’ end of the viral genome sequence and an *XbaI* linearization site [40]. 300 ng of the *XbaI*-linearized plasmid was used as template for RiboMAX T7 RNA polymerase (Promega) *in vitro* transcription. CHIKV strain La Reunion (CHIKV-LR, obtained from the EVAg repository) [66] and YFV strain 17D [67] containing plasmids were linearized using *NotI* and *AflII* restriction sites, respectively. 1 µg of linearized DNA was used as template for cap-directed transcription using the SP6 mMESSAGE mMACHINE kit (Thermo Fisher Scientific). The RNA was purified using RNA Clean & Concentrator-25 (Zymo Research). The integrity of the transcripts was checked by running 1 µg RNA on a 1% agarose- 3.7% formaldehyde gel.

### RAP-RNA protocol (based on Engreitz et al. 2014 [23])

#### Probe design, synthesis and validation.

Each viral genome target sequence was amplified with a pair of primers producing a 90–120 bp amplicon. The forward primer was 5’ tagged with a T7 promoter sequence (5’- TAATACGACTCACTATAG- 3’) and the reverse primer was 5’-tagged with DP3 oligo complementary sequence (5’- CCGCTGGAAGTGACTGACAC- 3’). Amplicons were designed to meet an optimal GC content of around 50%. The PCR reaction was performed using Q5 High-Fidelity DNA polymerase (NEB) with an initial denaturation at 98°C for 30 seconds followed by 35 cycles as follows: 98°C 10 seconds, 67°C 10 seconds, 72°C 30 seconds. The PCR products were resolved and purified on 1% agarose gel using Zymo clean Gel DNA Recovery (Zymo Research). The purified DNA for each viral target sequence were pooled in equal molar mass ratios. An additional cleaning step was then performed using DNA Clean & Concentrator-5 kit (Zymo Research). 300 ng of the DNA pool was used as template for RiboMAX T7 RNA polymerase (Promega) in vitro transcription, following the manufacturer’s procedures. The RNA was purified using RNA Clean & Concentrator-25 (Zymo Research). 2.5 µg of RNA was retrotranscribed using SuperScript IV Reverse Transcriptase (Thermo Fisher Scientific) as follows: 2.5 µg of RNA, DP3 oligo 12.5 µM, dNTPs 0.5 mM each were placed at 65°C for 5 minutes and chilled down in ice for 1 minute. To 20 µL of the RNA mix 20 µL of reaction mix was added, prepared according to the manufacture’s procedure. The RT mix was incubated 10 minutes at 23°C, 1 hour at 51°C and 5 minutes at 85°C. The remaining RNA was eliminated by alkaline degradation 10 minutes at 75°C, adding NaOH to a final concentration of 0.1 M. The NaOH was neutralized using 1M acetic acid and the cDNA purified with DNA clean & Concentrator-5 columns (Zymo Research). The yield of purified cDNA probes pool was determined using NanoDrop (Thermo Fisher Scientific). To check the size and integrity of the probes pool, the cDNA was resolved on Novex TBE 6% gel (Thermo Fisher Scientific) and stained with SYBR Gold Nucleic Acid Stain (Thermo Fisher Scientific).

To determine the efficiency of hybridization of the cDNA probes pool, we set up an RNAse H cleavage assay as follows (based on [39]). Viral RNA and DNA pool probes were incubated in a 1:30 molar ratio in nuclease-free water. The mixture was heated to 95°C for 5 minutes and cooled down to 37°C for 15 minutes. To the RNA:probe mix, 10X reaction buffer pH 8.3 (Tris-HCl 500 mM, KCl 750 mM, MgCl_2_ 30 mM, DTT 100 mM) and 4U of RNAse H (Thermo Fisher Scientific) was added. The mix was then incubated for 1 hour at 37°C. The DNA:RNA complexes were next denatured for 1 minute at 85°C. The probes were degraded for 15 minutes at 37°C using 2U Turbo DNAse I (Thermo Fisher Scientific). The RNA was then purified using the Clean and Concentrator-5 kit (Zymo). The RNA was next diluted 1:1000 before running an RT-qPCR using Takara one-step RT-qPCR kit (Takara) and primers flanking the hybridization target sequence. The most efficient probes were then selected for synthesis by the PAN Biotechnology Facility of Stanford University (S2 Table). Each oligo carries a Biotin tag at the 5’ end.

#### Infection and psoralen crosslinking.

10^6^ Huh-7.5 cells per sample were infected with HCV J6/JFH1 clone 2 (MOI:1), CHIKV-LR (MOI:1) or YFV-17D (MOI:1) for 24 (YFV, CHIKV), or 72 (HCV) hours. The cells were then trypsinized and resuspended in 0.5 mg/mL AMT-PBS solution for 15 minutes on ice, protected from light. The cells were transferred to a 10 cm petri dish and crosslinked for 15 minutes under a UV source (Stratalinker 2400, Stratagene) at 365 nm. The crosslinked cells were recovered using a cell scraper and pelleted at 250 g for 5 minutes at 4°C. Each pellet was washed once in 1 mL ice cold PBS and resuspended in 500 µL of cell fractionation buffer (PARIS kit, Invitrogen) supplemented with 200 U/mL RNAsin Plus (Promega). The cells were gently resuspended and incubated for 5 minutes on ice. The sample was then pelleted for 5 minutes spinning at 500 g at 4°C. The cytoplasmic fraction (supernatant) was transferred to a clean tube and resuspended in 600 µL TRIzol Reagent (Thermo Scientific). The RNA was purified using RNA Clean & concentrator-25 (Zymo) according to the manufacturer’s instructions.

#### RNA antisense purification.

To isolate small RNAs, 5 µg RNA sample was treated with 10 U Turbo DNAse I for 20 minutes at 37°C. 2–3 µg of DNAse-treated RNA was then used for RNA antisense purification. To isolate long RNA interactors, 3 µg RNA sample was depleted for ribosomal RNA using the NEBNext rRNA Depletion kit (NEB). 200–300 ng of rRNA depleted RNA was then used for RNA antisense purification. 15 pmol of probes were incubated with the RNA sample for 3 minutes at 80°C and 1 minute on ice. Next, 300 µL of pre-heated (55°C) 1X LiCl Hybridization buffer (Tris-HCl pH 7.5 10 mM, 1 mM EDTA, 500 mM LiCL, 1% Triton-X 100, 0.2% SDS, 0.1% sodium deoxycholate) was added to the sample and incubated for 2 hours shaking 1200 rpm at 55°C. 200 µL of Dynabeads MyOne Streptavidin C1 per sample were washed 4X in Tris-HCl pH 7.5 10 mM and 1X in 500 µL LiCl Hybridization buffer. Beads were then resuspended in 50 µL LiCl hybridization buffer. The washed beads were then added to the RNA sample and incubated additional 30 minutes at 37°C shaking 1000 rpm. 10% of the hybridization mix was saved as input sample. The rest of the beads were collected by a quick spin and the beads were separated from the supernatant using a magnetic stand. Beads were next washed 3X with 250 µL of Low Stringency Washing buffer (1X SSPE, EDTA pH 8.0 1 mM, 0.1% SDS, 1% Igepal CA-630, 4M Urea) for 5 minutes at 58°C, 3X with 250 µL of High Stringency Washing buffer (0.1X SSPE, EDTA pH 8.0 0.1 mM, 0.1% SDS, 1% Igepal CA-630, 4M Urea) for 5 minutes at 58°C, and 2X in 500 µL Turbo DNase I buffer for 2 minutes at RT shaking at 1000 rpm. Beads were then resuspended in 50 µL of Turbo DNAse I reaction mix (containing 8 U of DNAse) and incubated 15 minutes at 37°C shaking at 1000 rpm. Beads were then spun down and the supernatant collected in a nuclease-free clean tube. The elution step was then repeated. RNA was purified using the RNA Clean up and Concentrator-5 kit (Zymo), and stored at -80°C. RNA cross-linking was reversed by irradiating the RNA for 5 minutes at 254 nm UV [24,68]. After reverse crosslinking, the RNA was used for RT-qPCR analysis or library prep using either of the CATS small-RNAseq-kit (Diagenode) for short RNA interactors, or the NEBnext Ultra II (directional, Chapter 4) for long RNA interactors. Libraries were sequenced on Illumina miSeq or NextSeq instruments.

#### Bioinformatic analysis of RAP-RNA data.

To align and annotate the RNA captured in RNA-RAP libraries, and to identify statistically enriched target genes and significant peaks on the host transcriptome, we set up a computational analysis pipeline as outlined in S4 Fig. All comparisons had a balanced design of the same number of replicates of infected and control (non-infected) samples (S3 Table). For quality control, FastQC and fastx toolkits were used, followed by alignment using bowtie or STAR aligner [69,70]. Repeat-masked sequences were filtered using RepeatMasker. HTSeq or Subread featureCounts were used for gene quantification [71,72], and differential gene analysis was done using DESeq2 or edgeR [73,74]. Piranha was used for peak calling, and differential peak analysis was done using DiffBind. Significantly enriched peaks were defined as FDR < 0.05. The specific tool parameters applied are given in S3 Table. The number of raw reads, quality filtered reads, and mapped reads to host and virus are listed in S4 Table. The raw data and processed table files were submitted to GEO under accession no. GSE309201.

#### Bioinformatic analysis of RNA-Seq data.

Analysis of mRNA-Seq was carried out by first filtering FASTQ reads such that 80% of the read contained a mean score of 20. Reads were then directly mapped to hg18 using Bowtie, allowing up to 2 mismatches and discarding reads with multiple hits. Mapped read coordinates were then intersected with a meta-transcript file of the longest isoform of every coding gene in RefSeq and then counted. The resulting reads per gene count, for each RNA-Seq replicate then underwent statistical analysis and quantification using EdgeR.

#### siRNAs reverse transfections.

2.5x10^5^ Huh-7.5 cells per sample were seeded in 6-well plates in a total volume of 2 mL DMEM with 10% FBS. 500 µL OptiMEM was combined with 2.5 µL RNAiMax (Thermo) and siRNA (sequences listed in S2 Table) to a final concentration of 10 nM. This mix was incubated 5 minutes at RT and added directly to the cells.

#### Plasmid forward transfections.

2.5x10^5^ Huh-7.5 cells per sample were seeded in 6-well plates in a total volume of 2 mL DMEM with 10% FBS 24 hours prior to transfection. Then 250 µL OptiMEM was combined with 5 µL Lipofectamine 2000 (Thermo), and in a second tube 250 µL OptiMEM was combined with 2 µg of DNA plasmid. After 5 minutes at RT, these were mixed and incubated for an additional 15 minutes before addition to the cells.

#### RT-qPCR for long and small RNAs.

TaqMan RT-qPCR was performed using the TaqMan Fast Virus 1-step Master Mix (Life Technologies) according to manufacturer’s instructions and with primers as given in S2 Table. Before addition of 2 µL RNA per sample, the master mix was incubated at 65°C for 5 minutes and cooled for 1 minute on ice. Cycling was performed for 30 minutes at 50°C, 5 minutes at 95°C, and 40 cycles of: 95°C for 15 seconds, 60°C for 30 seconds, and 60°C for 45 seconds.

SYBR Green RT-qPCR was performed using the One step primescript RT-PCR kit (Takara) according to manufacturer’s instructions. Before addition of 2 µL RNA per sample, the master mix was incubated at 65°C for 5 minutes and cooled for 1 minute on ice. Cycling was performed for 30 minutes at 42°C, 5 minutes at 95°C, and 40 cycles of: 95°C for 10 seconds, and 60°C for 45 seconds.

Semi-quantitative RT-PCR was performed using SuperScript III (Thermo) and random hexamers. 2 µL of 1:2 diluted cDNA and the Q5 High-Fidelity DNA polymerase (NEB) for 40 cycles as follows: 98°C for 10 seconds, 60°C for 10 seconds, and 72°C for 30 seconds. The PCR products were resolved on 2.5-3% agarose gel.

Small RNAs were retrotranscribed using the MiScript II kit (Qiagen) and the HiSpec buffer according to manufacturer’s procedures. The RT reaction was performed over 1 hour at 37°C follow by an inactivation step at 95°C for 5 minutes. The qPCR reaction was set up as follows: 2 µL of 1:2 diluted cDNA per sample, 10 µL of One Step SYBR RT-PCR buffer (2X),0.8 µL of Takara Enzyme mix, 0.8 µL of 10 µM diluted miRNA forward specific primer/universal primer for each target, and nuclease free water to a total volume of 20 µL. Before addition of RNA, the master mix was incubated at 65°C for 5 minutes and cool for 1 minute on ice. Cycling parameters were: 40 cycles of 95°C for 10 seconds, and 60°C for 45 seconds.

#### Infectivity assays.

For focus forming units (FFU) titration of HCV, 10^4^ Huh-7.5 cells per well were seeded 24 hours before infection in 96-well plates. Samples to be titrated were diluted in 10-fold dilution series in triplicates and transferred to the cells. After 48 hours of infection, plates were fixed in methanol and washed twice in PBS-Tween 0.1%. The cells were incubated in 3% hydrogen peroxide for 5 minutes at RT, washed twice in PBS-Tween and incubated 10 minutes in PBS-K. The plates were incubated overnight at 4°C with 50 uL anti-NS5A antibody (9E10, kindly provided by Charles Rice) 1:10,000 in a humid chamber. Plates were washed twice with PBS-Tween and incubated with ECL anti-mouse IgG (Amersham, NAV931) 1:500 at 4°C overnight in a humid chamber or at RT for 1 hour. The secondary antibody was washed away twice with PBS-Tween and the cells were stained using DAB substrate 1–20 minutes at RT, while the development of staining was followed. Plates were rinsed twice in water and air-dried overnight. FFUs were automatically counted by Immunospot Count v5.2 Analyzer Pro Doc software.

For plaque forming unit (PFU) titration of YFV and CHIKV, 2x10^5^ BHK-21 cells were seeded in 6-well plates 24 hours prior to infection. 500 µL of virus containing supernatant were added to each well and plates were rocked every 15 minutes during a 1-hour incubation at 37 °C. 2X DMEM was mixed 1:1 with 2.4-4% Avicel (RC-581-NF) and FBS to a final concentration of 10% and pre-warmed at 37°C. 2 mL was applied to overlay each well and cells were incubated 48–72 hours without disturbing the plates. Plates were fixed with 3 mL per well of 7.4% formaldehyde and incubated 15 minutes at RT. The formaldehyde was removed and 1 mL per well of crystal violet 0.1% in water was added for 10 min. Plates were rinsed twice in water and air-dried overnight before counting of plaques.

#### Western blotting.

Cell pellets were resuspended in 1X RIPA buffer, and centrifuged at 4°C for 20 minutes at 12,500 rpm. Supernatants were collected in a clean tube and the protein concentration measured by BCA assay (Pierce, microplate BCA protein assay). 20–25 µg of protein lysate was used for immunoblot and compared to 8 µL of Precision Plus Protein Western C standard (Bio-Rad). Protein samples were resuspended in 4X LDS denaturing protein buffer and 10X NuPAGE reducing agent. Samples were pre-heated 10 minutes at 70°C before electrophoresis on 4–12% Bis-Tris Novex 1.0 mm gels in 1X MOPS running buffer for 30 minutes at 200V. Blotting was performed on ice using 1X NuPAGE Transfer buffer supplemented with 1 mL NuPAGE anti-oxidant reagent and 100 mL methanol. The membrane was incubated overnight in 5% milk-0.1% PBS-Tween with primary antibody (S2 Table) at 4°C rocking. After washing 3X in PBS-Tween at RT, the membrane was incubated 2 hours with the secondary antibody (S2 Table) at RT rocking. 1 µL Precision Protein Plus StrepTactin-HRP conjugated was added per 10 mL of milk-PBS-Tween. The ECL assay was performed using SuperSignal West Pico Chemiluminescent Substrate (Pierce) and the signal detected using a ChemiDoc (Bio-Rad).

#### KO cell lines.

*PTGES3* KO cell lines were generated by introduction of bi-allelic frameshift mutations after the ATG start codon using CRISPR-cas9 technology. A set of gRNAs targeting the *PTGES3* locus were cloned in pX459v2.0 using the pairs of oligos TS-O-1046/47 and TS-O-1048/49 (S2 Table). The pX459v2.0 plasmid was linearized using BbsI, and the resulting band was gel purified using Zymoclean Gel DNA Recovery, followed by an extra purification with DNA Clean & concentrator-5. The oligo pairs to generate gRNAs were annealed by heating the oligo mix for 5 minutes at 95°C and cooling down for 15 minutes at RT. 100 ng of BbsI-linearized plasmid were ligated with the correspondent oligo mix using T4 ligase (NEB). The resulting plasmids were used to chemically transform competent cells, plated on Ampicillin-containing selective LB-agar petri dishes. Clones were screened by sanger sequencing.

For generation of KO cells, 4x10^5^ Huh-7.5 cells per sample were plated in 6-well plates in 2 mL DMEM medium with 10% FBS 24 hours before transfection. 3 µg of plasmids were transfected with 8 µL of Lipofectamine 2000 (Thermo Fisher Scientific) according to manufacturer’s procedures. 24 hours later, selection with puromycin (5 µg/mL) was initiated for an additional 2 days. After single cell-cloning, resistant cells were screened for presence of the targeted protein by immunoblot for PTGES3 as described above.

For analysis of HCV replication and virus production, Huh-7.5 cells and the two PTGES3 knockout cell clones (#1 and #6) were seeded in 24-well plates at 20,000 cells/well with adjusted seeding densities to account for slower growth of knockout cells. Prior to infection, cells were counted and inoculated with J6/JFH1-c2 virus at MOI = 0.5 adjusted to viable cell counts. Infected cells were incubated for 72 at 37 °C, 5% CO_2_. Supernatants were collected and stored at −80°C until further analysis. Cells were trypsinized, washed twice with PBS, pelleted by centrifugation (500 × g, 5 min, 4 °C), and stored at −80°C for downstream analyses.

Intracellular RNA production was quantified by RNA extraction from cell pellets using the QIAwave RNA Plus Mini Kit (Qiagen) with an additional dry spin before elution. Viral RNA levels were quantified by TaqMan RT-qPCR (TaqMan Fast Virus kit, Thermo Fisher Scientific, 20 µl reactions) using 0.5 µM primers and probes as listed in S2 Table. Cycling was performed with the following conditions; reverse transcription at 50 °C for 30 min, polymerase inactivation at 95 °C for 5 min, followed by 40 amplification cycles of 20 sec at 95°C followed by 75 seconds at 60°C. Relative HCV RNA levels were calculated using RPS11 as the endogenous reference gene for normalization.

For infectivity titration, Huh-7.5 cells were seeded in 96-well plates (7,000 cells/well) and infected with serial dilutions (10 ⁻ ¹–10 ⁻ ³) of collected supernatants, followed by 48 h incubation at 37 °C. Plates were stained and fixed as described above. HCV infection was then quantified by automated FFU counting using an ImmunoSpot Series 5 UV Analyzer (Cellular Technology Ltd.). Plates were scanned using ImmunoSpot software (version 5.2) in clear-plate mode with automated focusing and exposure adjustment. FFUs were quantified using BioSpot software with diffuse object detection parameters optimized using negative controls and representative infected wells. Identical counting parameters were applied across all wells within each plate, and artefacts were excluded during quality control where necessary.

#### siRNA screen and FFU titration.

For the siRNA screen, reverse transfections were performed in 96-well plates using Lipofectamine RNAiMAX (Thermo Fisher Scientific). Dharmacon ON-TARGETplus siRNA pools including a non-targeting control pool (Horizon Discovery/Dharmacon) used for this study are listed in S2 Table. siRNA-lipid complexes were prepared in OptiMEM to yield a final siRNA concentration of 20 nM and 0.2 µL RNAiMAX per well. Huh-7.5 cells were then seeded directly onto the complexes at 7 × 10³ cells per well in 100 µL DMEM and incubated at 37 °C for 24h prior to viral infection. Transfection medium was removed 24 h post-transfection and replaced with 100 µL infection medium per well, consisting of DMEM supplemented with 10% FBS, 1% PenStrep and HCV J6/JFH1-c2 adjusted to a final concentration of 1.5 × 10³ FFU/mL determined to yield ~100 FFUs in pilot experiments. Mock-infected wells received virus-free medium. Cells were incubated for 48 h at 37 °C prior to analysis. Cell viability was assessed using the CyQUANT XTT Cell Viability Assay (Thermo Fisher Scientific) according to the manufacturer’s instructions. Briefly, 70 µL XTT reaction mixture was added per well and incubated for 4 h at 37 °C, followed by absorbance measurement at 450 nm with reference subtraction at 660 nm. For FFU measurements plates were fixed, stained and quantified as described above.

## Supporting information

S1 Fig Global identification of HCV interactors. Data related to Figs 1 and 3. **A.** RNAse H assay for identification of efficient probes. Probe hybridization was assessed by RT-qPCR based quantification of target abundancy after annealing and incubation with RNAse H compared to controls incubated without RNAse H. **B.** RNA abundance (TPM: transcripts per million) during HCV infection as a function of RAP log_2_ fold change enrichment in HCV infected compared to uninfected samples. Red dots indicate genes meeting significance thresholds of FDR < 0.05 and log_2_FC>2. **C.** Comparison of translation efficiency (TE; Geometric mean of read counts for RiboSeq/RNA-seq) among RAP enriched RNAs compared to non-enriched RNAs. Data from [43], grouped by HCV RNA RAP enrichment. *Mann-Whitney U-test was used for statistics.*
(TIFF)

S2 Fig miRNA antagomir targeting and expression levels of miRNA genes during LNA inhibitor treatment. **A.** Coverage plot of HCV RAP-RNA reads on pre-miR-17. Location of miR-17-5p and -3p are indicated in pink. Locked nucleic acid (LNA) inhibitors designed as antagomirs (green) and the miR-17-3p inhibitor (orange) are indicated. **B-C.** Relative expression levels of pri-forms of (B) miR-17 and (C) miR-122 (miRNA genes) are shown after treatment with the indicated LNA inhibitors. NT: Non-targeting control.
(TIFF)

S3 Fig XTT cytotoxicity assay for siRNA knock-down of selected host factors. Related to Fig 5J. ANOVA with Dunnett correction for multiple testing was used for statistics. ns: non-significant, *: p < 0.033, **: p < 0.002, ***: p < 0.0002, ****: p < 0.0001.
(TIFF)

S4 Fig Computational pipeline used for RAP-RNA analysis. (TIFF)

S1 Table Significant interactors from RAP-RNA. (XLSX)

S2 Table Details on RAP probe sequences, primer sequences, siRNA sequences and antibodies. (XLSX)

S3 Table Bioinformatic tool parameters applied in the analysis. (XLSX)

S4 Table Read counts from RAP-RNA analysis. (XLSX)

## References

[1] Welch MD. Why should cell biologists study microbial pathogens?. Mol Biol Cell. 2015;26(24):4295–301. doi: 10.1091/mbc.E15-03-0144 26628749 PMC4666125

[2] Teng M, Xia ZJ, Lo N, Daud K, He HH. Assembling the RNA therapeutics toolbox. Med Rev (2021). 2024;4(2):110–28. doi: 10.1515/mr-2023-0062 38680684 PMC11046573

[3] Damas ND, Fossat N, Scheel TKH. Functional Interplay between RNA Viruses and Non-Coding RNA in Mammals. Noncoding RNA. 2019;5(1):7. doi: 10.3390/ncrna5010007 30646609 PMC6468702

[4] Bartel DP. Metazoan MicroRNAs. Cell. 2018;173(1):20–51. doi: 10.1016/j.cell.2018.03.006 29570994 PMC6091663

[5] Trobaugh DW, Gardner CL, Sun C, Haddow AD, Wang E, Chapnik E, et al. RNA viruses can hijack vertebrate microRNAs to suppress innate immunity. Nature. 2014;506(7487):245–8. doi: 10.1038/nature12869 24352241 PMC4349380

[6] Jopling CL, Yi M, Lancaster AM, Lemon SM, Sarnow P. Modulation of hepatitis C virus RNA abundance by a liver-specific MicroRNA. Science. 2005;309(5740):1577–81. doi: 10.1126/science.1113329 16141076

[7] Sarnow P, Sagan SM. Unraveling the mysterious interactions between hepatitis C virus RNA and liver-specific MicroRNA-122. Annual Review of Virology. 2016;3(1):309–32. doi: 10.1146/annurev-virology-110615-042409 27578438

[8] Henke JI, Goergen D, Zheng J, Song Y, Schüttler CG, Fehr C, et al. microRNA-122 stimulates translation of hepatitis C virus RNA. EMBO J. 2008;27(24):3300–10. doi: 10.1038/emboj.2008.244 19020517 PMC2586803

[9] Shimakami T, Yamane D, Jangra RK, Kempf BJ, Spaniel C, Barton DJ, et al. Stabilization of hepatitis C virus RNA by an Ago2-miR-122 complex. Proc Natl Acad Sci U S A. 2012;109(3):941–6. doi: 10.1073/pnas.1112263109 22215596 PMC3271899

[10] Sedano CD, Sarnow P. Hepatitis C virus subverts liver-specific miR-122 to protect the viral genome from exoribonuclease Xrn2. Cell Host Microbe. 2014;16(2):257–64. doi: 10.1016/j.chom.2014.07.006 25121753 PMC4227615

[11] Li Y, Yamane D, Lemon SM. Dissecting the roles of the 5’ exoribonucleases Xrn1 and Xrn2 in restricting hepatitis C virus replication. J Virol. 2015;89(9):4857–65. doi: 10.1128/JVI.03692-14 25673723 PMC4403451

[12] Scheel TKH, Luna JM, Liniger M, Nishiuchi E, Rozen-Gagnon K, Shlomai A, et al. A Broad RNA virus survey reveals both miRNA dependence and functional sequestration. Cell Host Microbe. 2016;19(3):409–23. doi: 10.1016/j.chom.2016.02.007 26962949 PMC4826034

[13] Kokkonos KG, Fossat N, Nielsen L, Holm C, Hepkema WM, Bukh J, et al. Evolutionary selection of pestivirus variants with altered or no microRNA dependency. Nucleic Acids Res. 2020;48(10):5555–71. doi: 10.1093/nar/gkaa300 32374844 PMC7261151

[14] Sleiman D, Goldschmidt V, Barraud P, Marquet R, Paillart J-C, Tisné C. Initiation of HIV-1 reverse transcription and functional role of nucleocapsid-mediated tRNA/viral genome interactions. Virus Res. 2012;169(2):324–39. doi: 10.1016/j.virusres.2012.06.006 22721779

[15] Matsui M, Corey DR. Non-coding RNAs as drug targets. Nat Rev Drug Discov. 2017;16(3):167–79. doi: 10.1038/nrd.2016.117 27444227 PMC5831170

[16] Chi SW, Zang JB, Mele A, Darnell RB. Argonaute HITS-CLIP decodes microRNA-mRNA interaction maps. Nature. 2009;460(7254):479–86. doi: 10.1038/nature08170 19536157 PMC2733940

[17] Hafner M, Landthaler M, Burger L, Khorshid M, Hausser J, Berninger P, et al. Transcriptome-wide identification of RNA-binding protein and microRNA target sites by PAR-CLIP. Cell. 2010;141(1):129–41. doi: 10.1016/j.cell.2010.03.009 20371350 PMC2861495

[18] Mullari M, Lyon D, Jensen LJ, Nielsen ML. Specifying RNA-binding regions in proteins by peptide cross-linking and affinity purification. J Proteome Res. 2017;16(8):2762–72. doi: 10.1021/acs.jproteome.7b00042 28648085

[19] Mullari M, Fossat N, Skotte NH, Asenjo-Martinez A, Humphreys DT, Bukh J, et al. Characterising the RNA-binding protein atlas of the mammalian brain uncovers RBM5 misregulation in mouse models of Huntington’s disease. Nat Commun. 2023;14(1):4348. doi: 10.1038/s41467-023-39936-x 37468457 PMC10356804

[20] Castello A, Fischer B, Eichelbaum K, Horos R, Beckmann BM, Strein C, et al. Insights into RNA biology from an atlas of mammalian mRNA-binding proteins. Cell. 2012;149(6):1393–406. doi: 10.1016/j.cell.2012.04.031 22658674

[21] Castello A, Fischer B, Frese CK, Horos R, Alleaume A-M, Foehr S, et al. Comprehensive identification of RNA-Binding domains in human cells. Mol Cell. 2016;63(4):696–710. doi: 10.1016/j.molcel.2016.06.029 27453046 PMC5003815

[22] McHugh CA, Chen C-K, Chow A, Surka CF, Tran C, McDonel P, et al. The Xist lncRNA interacts directly with SHARP to silence transcription through HDAC3. Nature. 2015;521(7551):232–6. doi: 10.1038/nature14443 25915022 PMC4516396

[23] Engreitz JM, Sirokman K, McDonel P, Shishkin AA, Surka C, Russell P, et al. RNA-RNA interactions enable specific targeting of noncoding RNAs to nascent Pre-mRNAs and chromatin sites. Cell. 2014;159(1):188–99. doi: 10.1016/j.cell.2014.08.018 25259926 PMC4177037

[24] Aw JG, Shen Y, Wilm A, Sun M, Lim XN, Boon KL, et al. In vivo mapping of eukaryotic RNA interactomes reveals principles of higher-order organization and regulation. Molecular Cell. 2016;62(4):603–17. doi: 10.1016/j.molcel.2016.04.028 27184079

[25] Ziv O, Gabryelska MM, Lun ATL, Gebert LFR, Sheu-Gruttadauria J, Meredith LW, et al. COMRADES determines in vivo RNA structures and interactions. Nat Methods. 2018;15(10):785–8. doi: 10.1038/s41592-018-0121-0 30202058 PMC6168409

[26] Liao K-C, Xie X, Sundstrom AKB, Lim XN, Tan KK, Zhang Y, et al. Dengue and Zika RNA-RNA interactomes reveal pro- and anti-viral RNA in human cells. Genome Biol. 2023;24(1):279. doi: 10.1186/s13059-023-03110-9 38053173 PMC10696742

[27] Ziv O, Price J, Shalamova L, Kamenova T, Goodfellow I, Weber F. The short- and long-range RNA-RNA interactome of SARS-CoV-2. Molecular Cell. 2020;80(6):1067-77.e5. doi: 10.1016/j.molcel.2020.11.004 33259809 PMC7643667

[28] Yang SL, DeFalco L, Anderson DE, Zhang Y, Aw JGA, Lim SY, et al. Comprehensive mapping of SARS-CoV-2 interactions in vivo reveals functional virus-host interactions. Nat Commun. 2021;12(1):5113. doi: 10.1038/s41467-021-25357-1 34433821 PMC8387478

[29] Wu T, Cheng AY, Zhang Y, Xu J, Wu J, Wen L, et al. KARR-seq reveals cellular higher-order RNA structures and RNA-RNA interactions. Nat Biotechnol. 2024;42(12):1909–20. doi: 10.1038/s41587-023-02109-8 38238480 PMC11255127

[30] Polaris Observatory HCV Collaborators. Global change in hepatitis C virus prevalence and cascade of care between 2015 and 2020: A modelling study. Lancet Gastroenterol Hepatol. 2022;7(5):396–415. doi: 10.1016/S2468-1253(21)00472-6 35180382

[31] Bukh J. The history of hepatitis C virus (HCV): Basic research reveals unique features in phylogeny, evolution and the viral life cycle with new perspectives for epidemic control. J Hepatol. 2016;65(1 Suppl):S2–21. doi: 10.1016/j.jhep.2016.07.035 27641985

[32] Janssen HLA, Reesink HW, Lawitz EJ, Zeuzem S, Rodriguez-Torres M, Patel K, et al. Treatment of HCV infection by targeting microRNA. N Engl J Med. 2013;368(18):1685–94. doi: 10.1056/NEJMoa1209026 23534542

[33] van der Ree MH, de Vree JM, Stelma F, Willemse S, van der Valk M, Rietdijk S, et al. Safety, tolerability, and antiviral effect of RG-101 in patients with chronic hepatitis C: A phase 1B, double-blind, randomised controlled trial. Lancet. 2017;389(10070):709–17. doi: 10.1016/S0140-6736(16)31715-9 28087069

[34] Scheel TKH, Rice CM. Understanding the hepatitis C virus life cycle paves the way for highly effective therapies. Nat Med. 2013;19(7):837–49. doi: 10.1038/nm.3248 23836234 PMC3984536

[35] Manns MP, Maasoumy B. Breakthroughs in hepatitis C research: From discovery to cure. Nat Rev Gastroenterol Hepatol. 2022;19(8):533–50. doi: 10.1038/s41575-022-00608-8 35595834 PMC9122245

[36] Bartenschlager R, Baumert TF, Bukh J, Houghton M, Lemon SM, Lindenbach BD, et al. Critical challenges and emerging opportunities in hepatitis C virus research in an era of potent antiviral therapy: Considerations for scientists and funding agencies. Virus Res. 2018;248:53–62. doi: 10.1016/j.virusres.2018.02.016 29477639

[37] de Souza WM, Lecuit M, Weaver SC. Chikungunya virus and other emerging arthritogenic alphaviruses. Nat Rev Microbiol. 2025;23(9):585–601. doi: 10.1038/s41579-025-01177-8 40335675

[38] Douam F, Ploss A. Yellow fever virus: Knowledge gaps impeding the fight against an old foe. Trends Microbiol. 2018;26(11):913–28. doi: 10.1016/j.tim.2018.05.012 29933925 PMC6340642

[39] Wassarman DA, Steitz JA. Structural analyses of the 7SK ribonucleoprotein (RNP), the most abundant human small RNP of unknown function. Mol Cell Biol. 1991;11(7):3432–45. doi: 10.1128/mcb.11.7.3432-3445.1991 1646389 PMC361072

[40] Catanese MT, Loureiro J, Jones CT, Dorner M, von Hahn T, Rice CM. Different requirements for scavenger receptor class B type I in hepatitis C virus cell-free versus cell-to-cell transmission. J Virol. 2013;87(15):8282–93. doi: 10.1128/JVI.01102-13 23698298 PMC3719822

[41] Luna JM, Scheel TK, Danino T, Shaw KS, Mele A, Fak JJ. Hepatitis C virus RNA functionally sequesters miR-122. Cell. 2015;160(6):1099–110. doi: 10.1016/j.cell.2015.02.025 25768906 PMC4386883

[42] Zhao H, Cai Z, Rao J, Wu D, Ji L, Ye R, et al. SARS-CoV-2 RNA stabilizes host mRNAs to elicit immunopathogenesis. Mol Cell. 2024;84(3):490-505.e9. doi: 10.1016/j.molcel.2023.11.032 38128540

[43] Gerresheim GK, Bathke J, Michel AM, Andreev DE, Shalamova LA, Rossbach O, et al. Cellular gene expression during hepatitis C virus replication as revealed by ribosome profiling. Int J Mol Sci. 2019;20(6):1321. doi: 10.3390/ijms20061321 30875926 PMC6470931

[44] van Rooij E, Kauppinen S. Development of microRNA therapeutics is coming of age. EMBO Mol Med. 2014;6(7):851–64. doi: 10.15252/emmm.201100899 24935956 PMC4119351

[45] Reiss S, Harak C, Romero-Brey I, Radujkovic D, Klein R, Ruggieri A, et al. The lipid kinase phosphatidylinositol-4 kinase III alpha regulates the phosphorylation status of hepatitis C virus NS5A. PLoS Pathog. 2013;9(5):e1003359. doi: 10.1371/journal.ppat.1003359 23675303 PMC3649985

[46] Marceau CD, Puschnik AS, Majzoub K, Ooi YS, Brewer SM, Fuchs G, et al. Genetic dissection of Flaviviridae host factors through genome-scale CRISPR screens. Nature. 2016;535(7610):159–63. doi: 10.1038/nature18631 27383987 PMC4964798

[47] Sherwood AV, Rivera-Rangel LR, Ryberg LA, Larsen HS, Anker KM, Costa R, et al. Hepatitis C virus RNA is 5’-capped with flavin adenine dinucleotide. Nature. 2023;619(7971):811–8. doi: 10.1038/s41586-023-06301-3 37407817 PMC7616780

[48] Szklarczyk D, Kirsch R, Koutrouli M, Nastou K, Mehryary F, Hachilif R, et al. The STRING database in 2023: protein-protein association networks and functional enrichment analyses for any sequenced genome of interest. Nucleic Acids Res. 2023;51(D1):D638–46. doi: 10.1093/nar/gkac1000 36370105 PMC9825434

[49] Helwak A, Kudla G, Dudnakova T, Tollervey D. Mapping the human miRNA interactome by CLASH reveals frequent noncanonical binding. Cell. 2013;153(3):654–65. doi: 10.1016/j.cell.2013.03.043 23622248 PMC3650559

[50] Grosswendt S, Filipchyk A, Manzano M, Klironomos F, Schilling M, Herzog M, et al. Unambiguous identification of miRNA:target site interactions by different types of ligation reactions. Mol Cell. 2014;54(6):1042–54. doi: 10.1016/j.molcel.2014.03.049 24857550 PMC4181535

[51] Moore MJ, Scheel TKH, Luna JM, Park CY, Fak JJ, Nishiuchi E, et al. miRNA-target chimeras reveal miRNA 3’-end pairing as a major determinant of Argonaute target specificity. Nat Commun. 2015;6:8864. doi: 10.1038/ncomms9864 26602609 PMC4674787

[52] Nguyen TD, Trinh TA, Bao S, Nguyen TA. Secondary structure RNA elements control the cleavage activity of DICER. Nat Commun. 2022;13(1):2138. doi: 10.1038/s41467-022-29822-3 35440644 PMC9018771

[53] Flores-Jasso CF, Arenas-Huertero C, Reyes JL, Contreras-Cubas C, Covarrubias A, Vaca L. First step in pre-miRNAs processing by human Dicer. Acta Pharmacol Sin. 2009;30(8):1177–85. doi: 10.1038/aps.2009.108 19654583 PMC4006688

[54] Liz J, Portela A, Soler M, Gómez A, Ling H, Michlewski G, et al. Regulation of pri-miRNA processing by a long noncoding RNA transcribed from an ultraconserved region. Mol Cell. 2014;55(1):138–47. doi: 10.1016/j.molcel.2014.05.005 24910097

[55] Boytz R, Keita K, Pawlak JB, Laurent-Rolle M. Flaviviruses manipulate mitochondrial processes to evade the innate immune response. Npj Viruses. 2024;2(1):47. doi: 10.1038/s44298-024-00057-x 39371935 PMC11452341

[56] Huang Z h, Du Y p, Wen J t, Lu B f, Zhao Y. snoRNAs: functions and mechanisms in biological processes, and roles in tumor pathophysiology. Cell Death Discovery. 2022;8(1):259. doi: 10.1038/s41420-022-01056-8 35552378 PMC9098889

[57] Bergeron D, Fafard-Couture É, Scott MS. Small nucleolar RNAs: Continuing identification of novel members and increasing diversity of their molecular mechanisms of action. Biochem Soc Trans. 2020;48(2):645–56. doi: 10.1042/BST20191046 32267490 PMC7200641

[58] Gokhale NS, McIntyre ABR, McFadden MJ, Roder AE, Kennedy EM, Gandara JA, et al. N6-Methyladenosine in Flaviviridae Viral RNA Genomes Regulates Infection. Cell Host Microbe. 2016;20(5):654–65. doi: 10.1016/j.chom.2016.09.015 27773535 PMC5123813

[59] Kim B, Arcos S, Rothamel K, Jian J, Rose KL, McDonald WH, et al. Discovery of widespread host protein interactions with the pre-replicated genome of CHIKV using VIR-CLASP. Mol Cell. 2020;78(4):624-640.e7. doi: 10.1016/j.molcel.2020.04.013 32380061 PMC7263428

[60] Baquero-Pérez B, Bortoletto E, Rosani U, Delgado-Tejedor A, Medina R, Novoa EM, et al. Elucidation of the epitranscriptomic RNA modification landscape of chikungunya virus. Viruses. 2024;16(6):945. doi: 10.3390/v16060945 38932237 PMC11209572

[61] Baquero-Pérez B, Yonchev ID, Delgado-Tejedor A, Medina R, Puig-Torrents M, Sudbery I, et al. N6-methyladenosine modification is not a general trait of viral RNA genomes. Nat Commun. 2024;15(1):1964. doi: 10.1038/s41467-024-46278-9 38467633 PMC10928186

[62] Saxena T, Tandon B, Sharma S, Chameettachal S, Ray P, Ray AR, et al. Combined miRNA and mRNA signature identifies key molecular players and pathways involved in chikungunya virus infection in human cells. PLoS One. 2013;8(11):e79886. doi: 10.1371/journal.pone.0079886 24278205 PMC3836776

[63] Matera AG, Wang Z. A day in the life of the spliceosome. Nat Rev Mol Cell Biol. 2014;15(2):108–21. doi: 10.1038/nrm3742 24452469 PMC4060434

[64] Sharma E, Sterne-Weiler T, O’Hanlon D, Blencowe BJ. Global mapping of human RNA-RNA interactions. Mol Cell. 2016;62(4):618–26. doi: 10.1016/j.molcel.2016.04.030 27184080

[65] Lu Z, Zhang QC, Lee B, Flynn RA, Smith MA, Robinson JT, et al. RNA duplex map in living cells reveals higher-order transcriptome structure. Cell. 2016;165(5):1267–79. doi: 10.1016/j.cell.2016.04.028 27180905 PMC5029792

[66] Tsetsarkin K, Higgs S, McGee CE, De Lamballerie X, Charrel RN, Vanlandingham DL. Infectious clones of Chikungunya virus (La Réunion isolate) for vector competence studies. Vector Borne Zoonotic Dis. 2006;6(4):325–37. doi: 10.1089/vbz.2006.6.325 17187566

[67] Bredenbeek PJ, Kooi EA, Lindenbach B, Huijkman N, Rice CM, Spaan WJM. A stable full-length yellow fever virus cDNA clone and the role of conserved RNA elements in flavivirus replication. J Gen Virol. 2003;84(Pt 5):1261–8. doi: 10.1099/vir.0.18860-0 12692292

[68] Aw JGA, Shen Y, Nagarajan N, Wan Y. Mapping RNA-RNA interactions globally using biotinylated psoralen. J Vis Exp. 2017;(123). doi: 10.3791/55255 28570509 PMC5608146

[69] Langmead B, Trapnell C, Pop M, Salzberg SL. Ultrafast and memory-efficient alignment of short DNA sequences to the human genome. Genome Biol. 2009;10(3):R25. doi: 10.1186/gb-2009-10-3-r25 19261174 PMC2690996

[70] Dobin A, Davis CA, Schlesinger F, Drenkow J, Zaleski C, Jha S, et al. STAR: ultrafast universal RNA-seq aligner. Bioinformatics. 2013;29(1):15–21. doi: 10.1093/bioinformatics/bts635 23104886 PMC3530905

[71] Putri GH, Anders S, Pyl PT, Pimanda JE, Zanini F. Analysing high-throughput sequencing data in Python with HTSeq 2.0. Bioinformatics. 2022;38(10):2943–5. doi: 10.1093/bioinformatics/btac166 35561197 PMC9113351

[72] Liao Y, Smyth GK, Shi W. featureCounts: An efficient general purpose program for assigning sequence reads to genomic features. Bioinformatics. 2014;30(7):923–30. doi: 10.1093/bioinformatics/btt656 24227677

[73] Love MI, Huber W, Anders S. Moderated estimation of fold change and dispersion for RNA-seq data with DESeq2. Genome Biol. 2014;15(12):550. doi: 10.1186/s13059-014-0550-8 25516281 PMC4302049

[74] Chen Y, Chen L, Lun ATL, Baldoni PL, Smyth GK. edgeR v4: powerful differential analysis of sequencing data with expanded functionality and improved support for small counts and larger datasets. Nucleic Acids Res. 2025;53(2):gkaf018. doi: 10.1093/nar/gkaf018 39844453 PMC11754124

