# Supplementary figures and images for: Characterization of the virus-host RNA-RNA interactome across important human pathogenic RNA viruses
Source: PLoS Pathog. 2026 May 15;22(5):e1014217. doi: 10.1371/journal.ppat.1014217 (PMC13215601; doi:10.1371/journal.ppat.1014217)

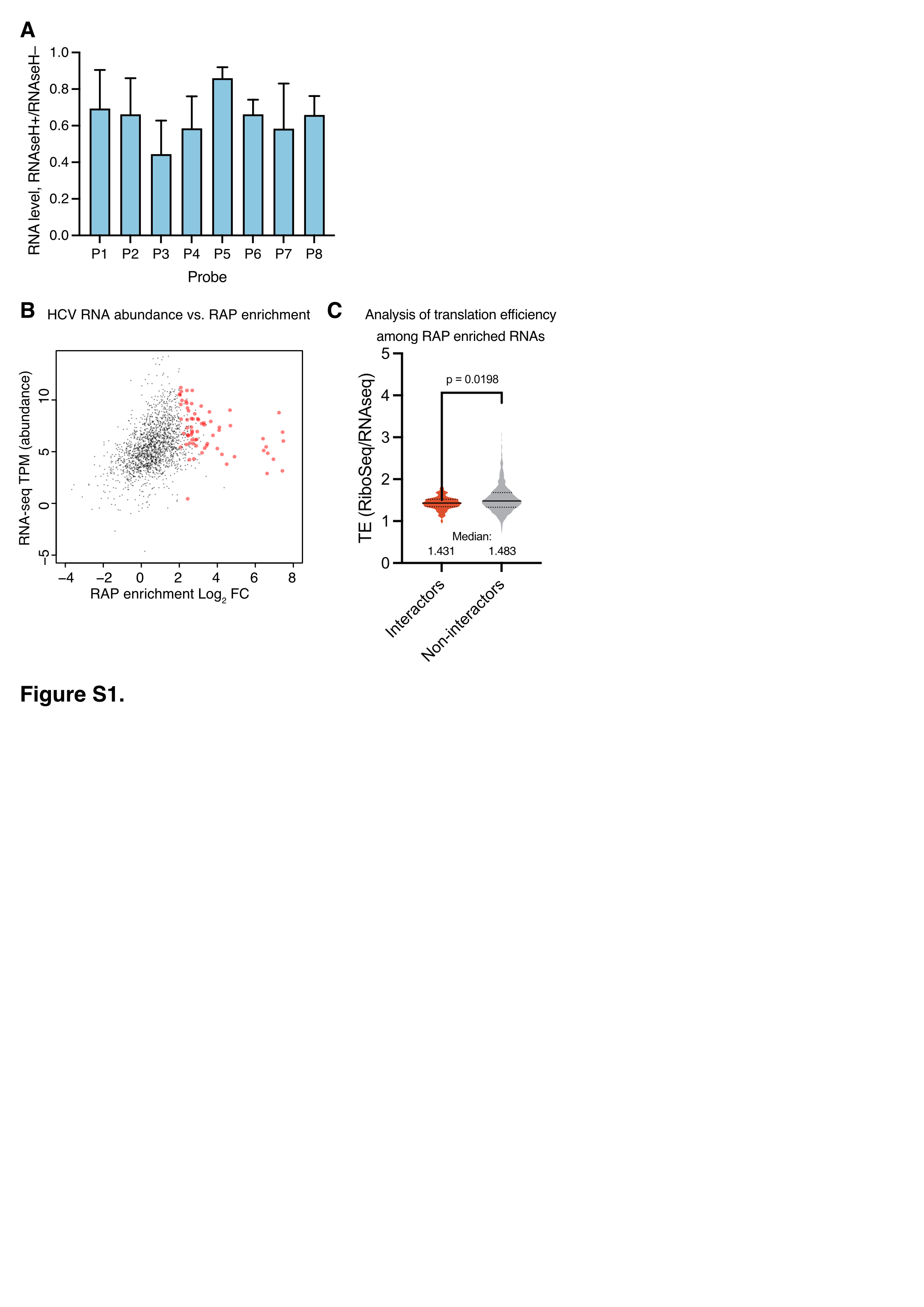

Supplement: S1 Fig — A. RNAse H assay for identification of efficient probes. Probe hybridization was assessed by RT-qPCR based quantification of target abundancy after annealing and incubation with RNAse H compared to controls incubated without RNAse H. B. RNA abundance (TPM: transcripts per million) during HCV infection as a function of RAP log2 fold change enrichment in HCV infected compared to uninfected samples. Red dots indicate genes meeting significance thresholds of FDR < 0.05 and log2FC>2. C. Comparison of translation efficiency (TE; Geometric mean of read counts for RiboSeq/RNA-seq) among RAP enriched RNAs compared to non-enriched RNAs. Data from [43], grouped by HCV RNA RAP enrichment. Mann-Whitney U-test was used for statistics. (TIFF) [file ppat.1014217.s001.tiff]

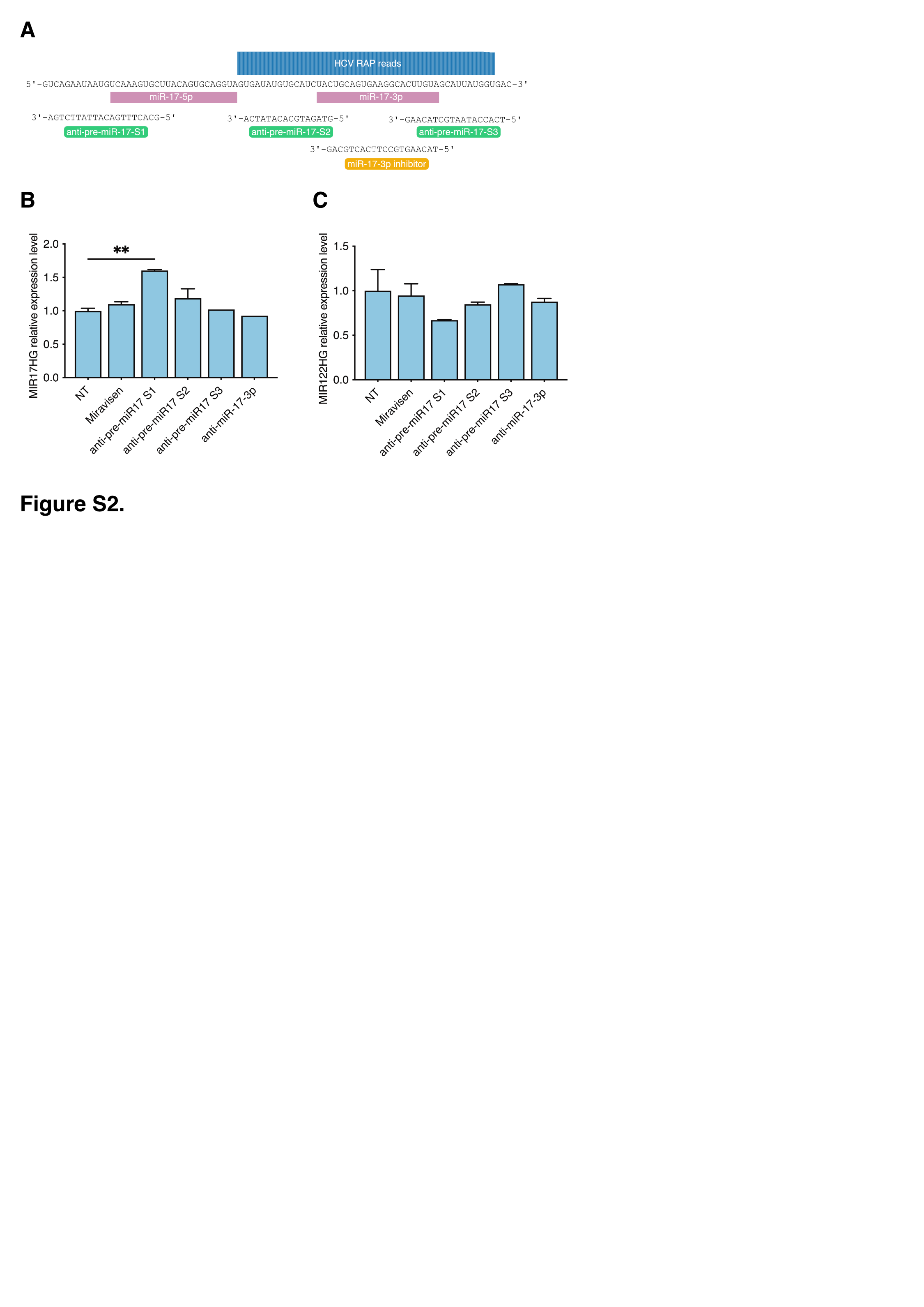

Supplement: S2 Fig — A. Coverage plot of HCV RAP-RNA reads on pre-miR-17. Location of miR-17-5p and -3p are indicated in pink. Locked nucleic acid (LNA) inhibitors designed as antagomirs (green) and the miR-17-3p inhibitor (orange) are indicated. B-C. Relative expression levels of pri-forms of (B) miR-17 and (C) miR-122 (miRNA genes) are shown after treatment with the indicated LNA inhibitors. NT: Non-targeting control. (TIFF) [file ppat.1014217.s002.tiff]

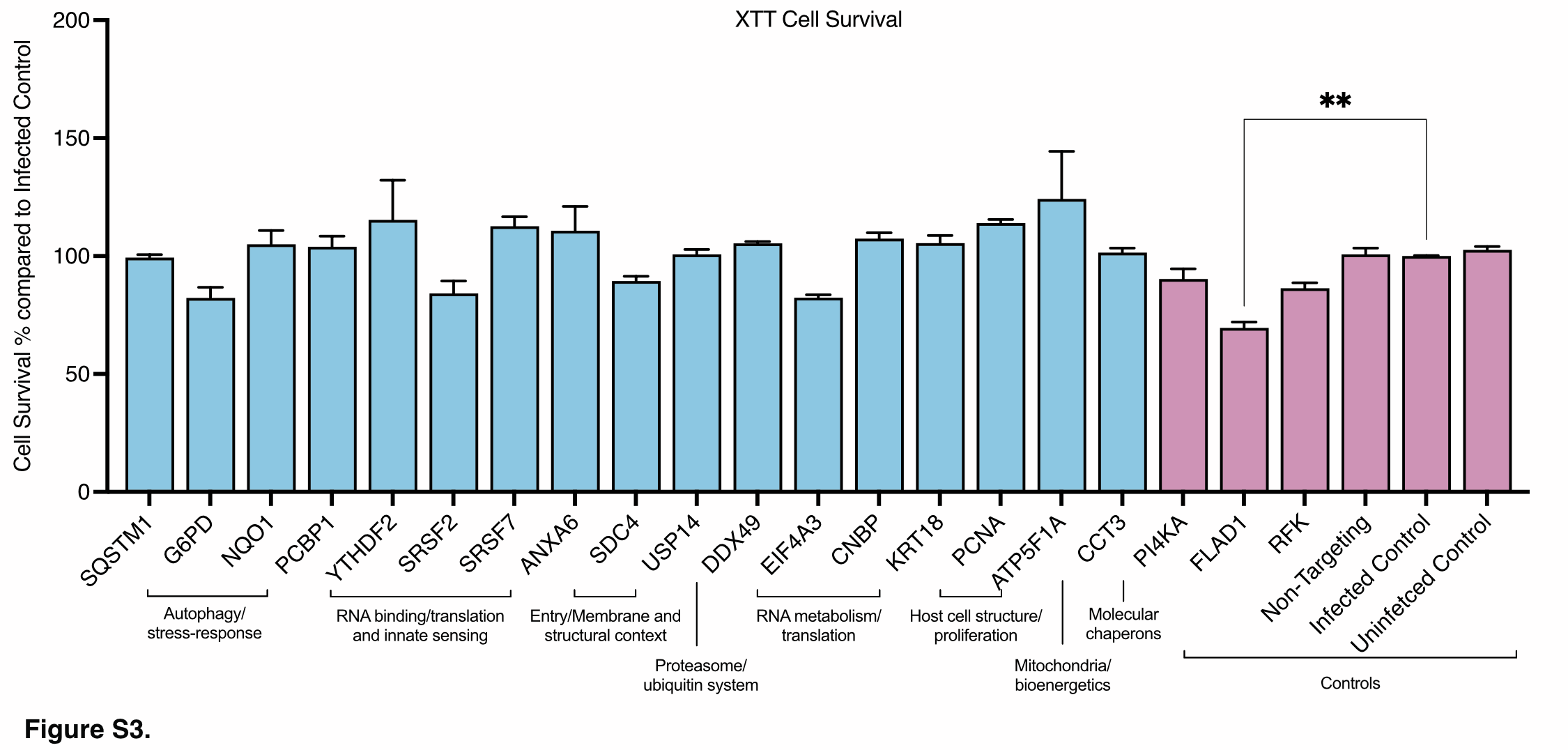

Supplement: S3 Fig — Related to Fig 5J. ANOVA with Dunnett correction for multiple testing was used for statistics. ns: non-significant, *: p < 0.033, **: p < 0.002, ***: p < 0.0002, ****: p < 0.0001. (TIFF) [file ppat.1014217.s003.tiff]

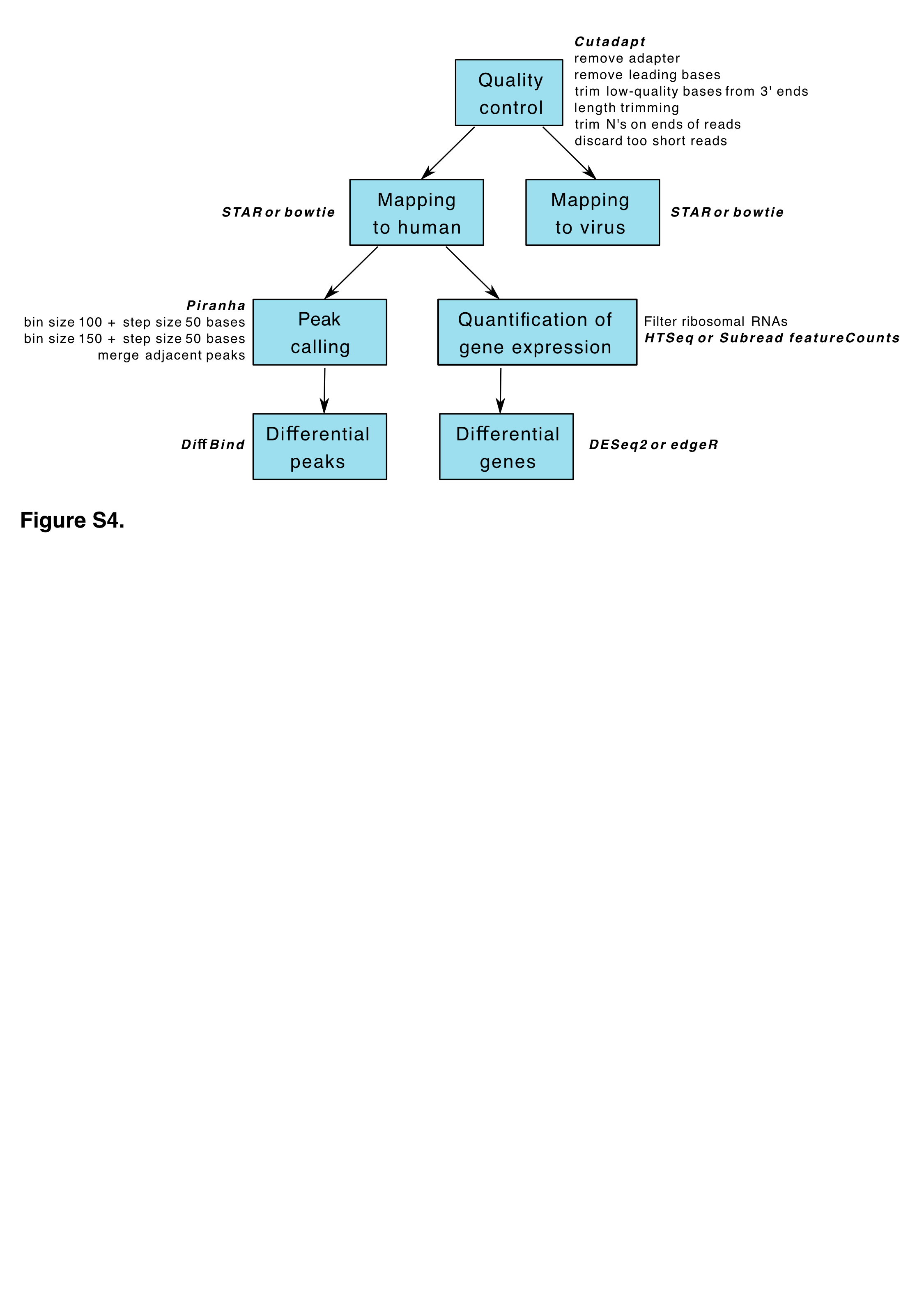

Supplement: S4 Fig — (TIFF) [file ppat.1014217.s004.tiff]
